# Chemometrically driven multiplexed metal ion detection using a triple emitting quantum dots–based nanoprobe

**DOI:** 10.1007/s00216-024-05661-7

**Published:** 2024-11-26

**Authors:** Rafael C. Castro, Ricardo N. M. J. Páscoa, M. Lúcia M. F. S. Saraiva, João L. M. Santos, David S. M. Ribeiro

**Affiliations:** LAQV, REQUIMTE, Laboratory of Applied Chemistry, Department of Chemical Sciences, Faculty of Pharmacy, University of Porto, Rua de Jorge Viterbo Ferreira nº 228, 4050-313 Porto, Portugal

**Keywords:** Chemometrics, Metal ions, Multi-emitter nanoprobe, Quantification, Discrimination, Second-order data

## Abstract

Metal ion pollution poses a global concern due to its significant risks to both human health and environmental well-being. The toxicity of these ions can increase when they coexist, interacting with each other and with other harmful substances, even at low concentrations. Therefore, an accurate, rapid, and cost-effective methodology is urgently needed for the simultaneous quantification of multiple metal ions. This study presents a new approach for the multiplexed detection of various metal ions (Ag^+^, Cu^2+^, Hg^2+^, Al^3+^, Pb^2+^, Fe^3+^, Fe^2+^, Zn^2+^, Ni^2+^, Cd^2+^, and Ca^2+^) using a triple-emission nanoprobe comprising carbon dots and distinctly capped CdTe quantum dots, specifically green-emitting glutathione -quantum dots and red-emitting 3-mercaptopropionic acid-quantum dots. The method achieved high accuracy by analysing first- and second-order photoluminescence data with distinct advanced chemometric tools. R^2^_P_ values for partial least squares and unfolded partial least square models exceeding 0.9 for several metal ions at low concentrations (mmol L^−1^) were obtained. Additionally, PL second-order data yielded significantly better results than PL first-order data, attributed to the distinct behaviour of the metal ions over time. Interestingly, it was also noted for the first time the significant contribution of the molar ratio between the metal ions on the models’ accuracy. This novel method provides a highly accurate and efficient way to detect multiple metal ions simultaneously, paving the way for improved environmental monitoring and pollution assessment. The utilization of the proposed method contributes to a better understanding of the complex interactions in mixed metal ion systems, allowing for earlier detection and mitigation of metal ion contamination threats.

**Graphical Abstract:**

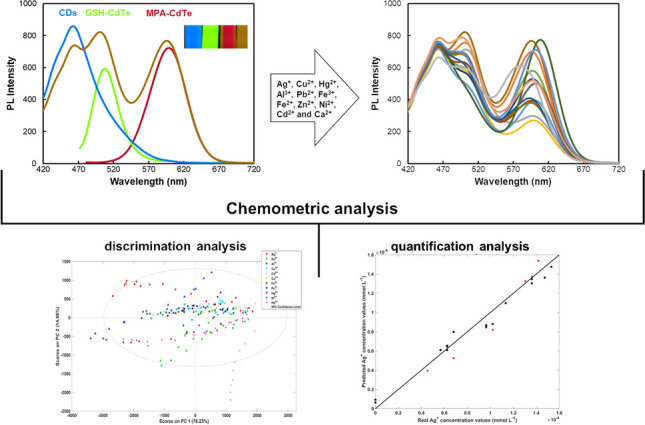

**Supplementary Information:**

The online version contains supplementary material available at 10.1007/s00216-024-05661-7.

## Introduction

With the increase of industrial development, metal ion pollution has evolved into a global concern, posing significant risks to both human and environmental well-being [1]. In contrast to other harmful substances, metal ions are not only difficult to transform into non-toxic forms but exhibit often high bioavailability, leading to their accumulation in the human body [1]. Even though certain metal ions play essential roles in human biological functions, they can lead to chronic or acute poisoning under conditions of elevated exposure. The issue of metal ion contamination is not confined to a specific type of metal ion but encompasses the coexistence of multiple metal ions within various environments [1, 2]. These ions can interact among themselves or with other harmful substances, giving rise to additive toxicity and synergistic mixture effects [3, 4]. Consequently, the existence of individual metal ions, even at low concentrations, can heighten the toxicity levels when other contaminants coexist in the same sample. For that reason, multiplexed detection of metal ions represents a transformative milestone in chemical analysis, providing the possibility of simultaneously identifying and quantifying multiple metal ions within a single analysis. This approach not only enhances the efficiency of analysis but also reduces both time and costs, offering distinct advantages compared to traditional single-target methods in the analysis of complex samples [5]. The simultaneous detection of multiple metal ions provides a more comprehensive analysis of complex samples, offering insights into the coexistence and interactions of different metal species [5]. Its significance extends to environmental monitoring, playing a crucial role in assessing soil contamination, water quality, and air pollution. Advanced and innovative multiplexed detection methods need to be developed and implemented to guarantee accurate and reliable assessments of unidentified samples, thereby supporting the capacity to effectively address diverse public health emergencies. Numerous recent endeavours have been focused on achieving concurrent detection of multiple heavy metal ions, employing various methodologies such as inductively coupled plasma mass spectrometry (ICP-MS) [6, 7], electrochemical techniques [8, 9], atomic absorption spectrometry (AAS) [10, 11], surface-enhanced Raman spectroscopy (SERS) [12], and X-ray fluorescence spectrometry (XRF) [13]. While each of these techniques possesses distinct advantages, ensuring reliable and accurate results, their implementation demands sophisticated and complex instruments, high associated costs, and the necessity for well-trained operators. Furthermore, these methods are labour-intensive and time-consuming, involving multiple lengthy procedures, making them less suitable for efficient screening applications [14].

Optical methodologies for multiplexed detection [15], particularly those utilizing photoluminescence (PL)-based approaches in combination with chemometric data analysis, have become a valuable complement to traditional multiplexed assays [16–19]. In this regard, photoluminescent nanocrystalline materials, such as semiconductor quantum dots (QDs) and carbon dots (CDs), stand out among various available fluorophores due to their superior optical and electronic properties, making them noteworthy elements in multiplexed detection approaches [20–22]. These properties include high brightness with enhanced sensitivity, resistance to photobleaching, which is particularly useful for prolonged measurements, broad absorption spectra enabling flexibility in excitation wavelength selection, possibility of composition and size-dependent spectral adjustments covering the entire visible and near-infrared spectrum, narrow emission bands facilitating the fitting of multiple signals, Gaussian-shaped emission bands aiding in the deconvolution of overlapping profiles, long PL lifetimes allowing temporal discrimination, and highly tunable surface chemistry for specific recognition of various analytical targets through biomolecule and polymer conjugation [2, 20–22].

An efficient multiplexed strategy that can be successfully explored is the development of multi-emitter nanoprobes comprising diverse QDs of varying size, nature, configuration, and/or composition, emitting at distinct wavelengths, which enables the acquisition of specific analyte-response profiles through multi-point detection [23–27]. The subsequent application of chemometric models to process the obtained PL responses facilitates the accurate and selective detection of multiple analytical targets in a single sample analysis [23–26]. These chemometric models provide a means to extract valuable information from the PL dataset, revealing relationships between samples and analysed variables, through mathematical and statistical methods, crucial for achieving accurate and precise multi-parametric analysis [16, 28].

In the development of innovative methodologies using QDs-based multi-emitter nanoprobes and chemometrics analysis, researchers face crucial decisions in selecting the type of data to be generated (whether it is zero-, first-, second-, or higher-order data) [16, 29]. This decision significantly influences the model selection, which, in turn, is determined by the research goals (discrimination or quantification) and the nature of the data, considering a linear or nonlinear behaviour [16, 30]. Increasing the complexity of the data collected per sample analysis is anticipated to yield more refined quantitative or qualitative analytical estimations, enhancing the overall figure of merits such as sensitivity and selectivity [31]. This means that higher data complexity allows for better qualitative discrimination of each metal ion and supports semi-quantitative analysis of metal ions in a given sample [32].

In this study, a triple-emission nanoprobe consisting of blue-emitting CDs and two distinctly capped CdTe QDs (green-emitting glutathione (GSH)-QDs and red-emitting 3-mercaptopropionic acid (MPA)-QDs) was utilized for the multiplexed detection of various metal ions (Ag^+^, Cu^2+^, Hg^2+^, Al^3+^, Pb^2+^, Fe^3+^, Fe^2+^, Zn^2+^, Ni^2+^, Cd^2+^, and Ca^2+^), with the objective of acquiring a broader variety of specific response profiles for each metal ion. The acquired PL dataset, comprising either first-order (spectral) or second-order (kinetic spectral) data, was analysed using appropriate chemometric models to quantify and/or discriminate distinct mixtures of metal ions. This study also aimed to compare which type of data, differing in complexity (first- or second-order data), provides more selective and accurate results. For discrimination purposes, partial least squares-discriminant analysis (PLS-DA) was employed, while PLS and unfolded-PLS (U-PLS) models were applied for quantification.

## Materials and methods

### Reagents and solutions

Every solution and standard utilized in this research were precisely prepared from analytical reagent-grade chemicals, without undergoing any prior treatment. The water utilized in these preparations underwent purification via a Milli-Q system, ensuring a conductivity level that did not surpass 0.1 µS cm^−1^.

Reagents in their powdered form, such as silver nitrate (AgNO_3_, > 99%), ferric chloride (FeCl_3_, 97%), ferrous sulphate heptahydrate (FeSO_4_.7H_2_O, ≥ 99.0%), zinc chloride (ZnCl_2_, ≥ 98%), nickel chloride (NiCl_2_, 99%), cadmium chloride (CdCl_2_, ≥ 99%), and calcium chloride (CaCl_2_, ≥ 97%), were procured from Sigma-Aldrich® (St. Louis, MO, USA). The other reagents employed as 1000 mg L^−1^ standard solutions, which include copper nitrate (Cu(NO_3_)_2_, in 0.5 mol L^−1^ HNO_3_), mercury nitrate (Hg(NO_3_)_2_, in 0.5 mol L^−1^ HNO_3_), aluminium nitrate (Al(NO_3_)_3_ in 0.5 mol L^−1^ HNO_3_), and lead nitrate (Pb(NO_3_)_2_ in 0.5 mol L^−1^ HNO_3_), were acquired from Merck® (Darmstadt, Germany).

Therefore, in the case of Ag^+^, Fe^2+^, Zn^2+^, Ni^2+^, Cd^2+^, and Ca^2+^, a 0.8 mmol L^−1^ intermediate solution of the metal ion was prepared by dissolving an appropriate amount of the respective powder in 50 mL of deionized water, except for Fe^3+^, which was prepared in 50 mL of 0.1 mol L^−1^ HNO_3_. For the Cu^2+^, Hg^2+^, Al^3+^, and Pb^2+^, the 0.8 mmol L^−1^ intermediate solution was prepared through proper dilution from the respective standard solution in 50 mL of deionized water.

The synthesis of MPA-CdTe QDs followed the conventional hydrothermal synthesis route, utilizing the two-pot approach proposed by Zou et al. [33]. Some modifications were introduced to this method, as detailed in our previous works [34–36]. The molar ratios of Cd:Te:capping ligand were set at 1:0.1:1.7 for MPA, maintaining a pH of 11.5. For the GSH-capped CdTe QDs, the hydrothermal one-step synthetic route proposed by Qian [37] was employed with a few adjustments outlined in refs. [38, 39]. The experimental conditions included a Cd:Te:GSH molar ratio of 1:0.2:1.2, and the pH was maintained at 10.5. The obtained CdTe QDs underwent precipitation in absolute ethanol (C_2_H_5_OH, 99.8%, Panreac, Barcelona, Spain) to eliminate excess reactants. The resulting precipitates were isolated through centrifugation. Subsequently, the nanomaterials were subjected to vacuum drying, stored in amber flasks, and protected from light. The QDs’ intermediate solutions were prepared by dissolving an appropriate amount of the corresponding precipitates in deionized water.

To synthesize carbon dots (CDs), the aqueous synthetic route protocol was followed as outlined in our previous works [40, 41]. The resulting crude solution underwent a purification process through dialysis against Milli-Q water for a period of 5 days, utilizing a dialysis membrane (Spectra/Por 6–1000 MWCO, Spectrum Labs™). The intermediary solutions of CDs were daily prepared by suitable dilution from the purified solution.

### Equipment

The pH control during the synthesis process of QDs was achieved using a sensION + pH31 GLP Laboratory pH meter. A refrigerated centrifuge (Thermo Electron Jouan BR4I, Waltham MA, USA) was used for the separation of precipitated QDs.

The measurements of the absolute photoluminescence quantum yields (PL QY) for all nanomaterials were carried out by Quantaurus QY C11347-11 spectrometer (Hamamatsu, Japan) equipped with an integrating sphere. A Lifetime spectrofluorometer (DeltaFlexTM TCSPC, Horiba Scientific, Kyoto, Japan), equipped with a PPD picosecond detection module, NanoLED pulsed light source, and DeltaHub timing electronics was utilized for PL lifetime measurements of all synthesized nanomaterials. A LUDOX® scattering solution served as a lifetime reference, facilitating the calibration of the instrument response function (IRF) for the lifetime curve.

For optical characterization and PL assays, PL emission spectra of the nanoparticles were collected using a Jasco FP-6500 spectrofluorometer (Easton, MD, USA).

### Fluorometric measurement procedure

To conduct the PL measurements, the GSH-CdTe intermediate solution was prepared by dissolving 8.5 mg of the purified precipitate in 15 mL of deionized water, resulting in a mass concentration of 0.57 mg mL^−1^. In the case of MPA-CdTe intermediate solution, 9.2 mg of the powder was dissolved in 10 mL of deionized water, achieving a concentration of 0.92 mg mL^−1^ (w/v). For the CDs’ intermediary solution, a dilution ratio of 1:1500 (v/v) from the crude solution was performed (100 µL diluted in 150 mL of deionized water).

In the fluorometric procedure, the required amounts of metal ions, either individually or in a mixture, were added initially to Eppendorf tubes, followed by the addition of the appropriate volume of deionized water to reach a final volume of 2000 µL.

Afterwards, sequential additions were made, including 80 µL of CD intermediate solution, 20 µL of MPA-CdTe intermediate solution, 10 µL of GSH-CdTe intermediate solution, and 1890 µL of the previously mentioned metal ion solution, reaching a final volume of 2000 µL.

Immediately after adding the metal ion solution, the mixture was stirred, and the emission spectra were measured in a quartz cell with a 10-mm optical path. The emission spectra were collected at a fixed excitation wavelength of 400 nm, using slit widths of 5.0 nm for both excitation and emission. Measurements were recorded every minute for up to 5 min, covering an emission wavelength range from 420 to 750 nm, with 1-nm interval. The first-order data considered only the emission wavelength range from 420 to 750 nm at time zero, yielding a total of 331 data points. The second-order data was obtained over the same emission wavelength range, measured every minute for the first 5 min, resulting in 1986 data points (331 × 6).

### Chemometric models for data analysis

The chemometric analysis was conducted using distinct models. Principal component analysis (PCA) [42] assisted in identifying outliers based on the analysis of squared residual statistics and Hoteling’s T^2^ statistics graph, as well as on the visualization of clusters. Partial least squares (PLS) [43] regression was employed to establish quantification models relating PL first-order data to metal concentrations. Partial least squares-discriminant analysis (PLS-DA) [44] was used for discrimination analysis, specifically to distinguish the presence of metal ions in the samples based on PL first-order data. Unfolded partial least squares (U-PLS) was used to establish quantification models relating PL second-order data to metal concentrations [45]. All fluorescence data were mean-centred before applying any of these chemometric tools.

For the application of PLS U-PLS and PLS-DA models, the PL data were divided into two datasets: one with 70% of data used for calibration and another with 30% of data used for validation. The division of the data was performed randomly for all models, ensuring that certain criteria were met. For PLS and U-PLS, this division guaranteed that the values of the metal ions included in the validation set fell within the range of those present in the calibration set. For PLS-DA, this division ensured an equal distribution of samples in each category to prevent imbalanced categories [46].

Subsequently, the calibration dataset was employed to optimize PLS and PLS-DA models by selecting the optimal number of latent variables (LVs) and determining the importance of different spectral regions. Spectral region optimization aimed to determine which QD of the triple-emission nanoprobe was more important for each quantification/discrimination. In this context, the PL data were divided into three different spectral regions, considering the PL emission profile of each QD: spectral region R1 within 420 and 474 nm, which corresponds to the CD emission; spectral region R2 encompassing 475 to 544 nm, which corresponds to the GSH-CdTe emission; and spectral region R3 from 545 to 750 nm, which corresponds to the MPA-CdTe emission. The optimization in terms of spectral region was performed only using the data of individual metal ions, with the findings applied to the binary and ternary mixtures of metal ions and U-PLS application. The leave-one-sample-out cross-validation method was used to assess the optimal number of LVs for both PLS and PLS-DA models. The optimal number of LVs for PLS and U-PLS models was determined based on the Haaland and Thomas criterion [47]. In essence, this criterion suggests that the optimal number of LVs should be determined based on the prediction error sum of squares (PRESS). More precisely, the ideal LV count is identified when the PRESS values are not significantly different (F-ratio probability below 0.75) from the minimum PRESS value obtained using a greater number of LVs. The PRESS value measures the effectiveness of PLS models in estimating concentrations. For PLS-DA models, the optimal number of LVs was determined by balancing the lowest number of LVs with the highest percentage of correct predictions obtained through cross-validation.

To select the best calibration models for PLS, a compromise was sought between minimizing the number of LVs, minimizing the root mean square error of calibration (RMSEC) and cross-validation (RMSECV), and maximizing the coefficients of determination for calibration (R^2^_C_) and cross-validation (R^2^_CV_). To determine the optimal calibration model for PLS-DA, a compromise was pursued between minimizing the number of LVs and maximizing the number of correct predictions, which were obtained by adding the diagonal values of the confusion matrices. This analysis was conducted considering only the calibration models. The best PLS and U-PLS models for binary and ternary metal ion mixtures were selected based on an analysis that considered each metal ion individually across the entire spectral region. Further details will be provided.

The PLS and U-PLS models were developed by considering each chemical parameter individually, using the PLS-1 algorithm. The PLS-DA models, on the other hand, were constructed by considering all chemical parameters together, using the PLS-2 algorithm. The accuracy assessment of the optimized calibration models involved projecting the validation set onto these models. For PLS models, the root mean square error of prediction (RMSEP) and coefficient of determination of prediction (R^2^_P_) parameters were used to assess the accuracy of the models. For PLS-DA models, accuracy was assessed by the percentage of correct predictions using the validation set.

The following equation was used to calculate the RMSEC, RMSECV, and RMSEP:

$$\text{RMSE}=\sqrt{\frac{\sum_{i=1}^{N}{\left( {\widehat{y}}_{i}-{y}_{i}\right)}^{2}}{N}}$$

where ${\widehat{y}}_{i}$ is the corresponding value obtained for sample $i$ for calibration (RMSEC), cross-validation (RMSECV), and prediction (RMSEP); ${y}_{i}$ corresponds to the experimental value for sample $i$; and $N$ represents the number of samples.

In the case of binary and ternary metal ion mixtures, the accuracy of PLS and U-PLS models was evaluated based on RMSECV, RMSEP, R^2^_CV_, and R^2^_P_.

The PCA, PLS, and PLS-DA models and respective calculations were performed in Matlab 2023a environment (MathWorks, Natick, MA, USA), using the PLS Toolbox version 9.2.1 (Eigenvector Research Inc., Wenatchee, WA, USA). For U-PLS modelling, the MVC2 interface (available for download at [48]) was used under the Matlab environment.

## Results and discussion

### Optical properties of the synthesized QDs

In the development of a PL probe designed for the quantification and/or differentiation of multiple metal ions, a combined nanoprobe emitting at multiple wavelengths was assessed. This experiment involved testing the combination of QDs with diverse compositions and sizes in a three-emission probe. The inclusion of QDs with specific affinities for distinct metal ions allows for multi-point detection, furnishing supplementary and valuable information for accurate analyte determination. Ideally, the QD emitters should be combined without substantial spectral overlap, resulting in a combined nanoprobe with spectrally resolved QDs emitters. Three QDs with dissimilar colour emissions were evaluated, namely blue-emitted carbon dots, green-emitted glutathione (GSH) CdTe QDs, and red-emitted 3-mercaptopropionic acid (MPA) CdTe QDs.

**Fig. 1:**
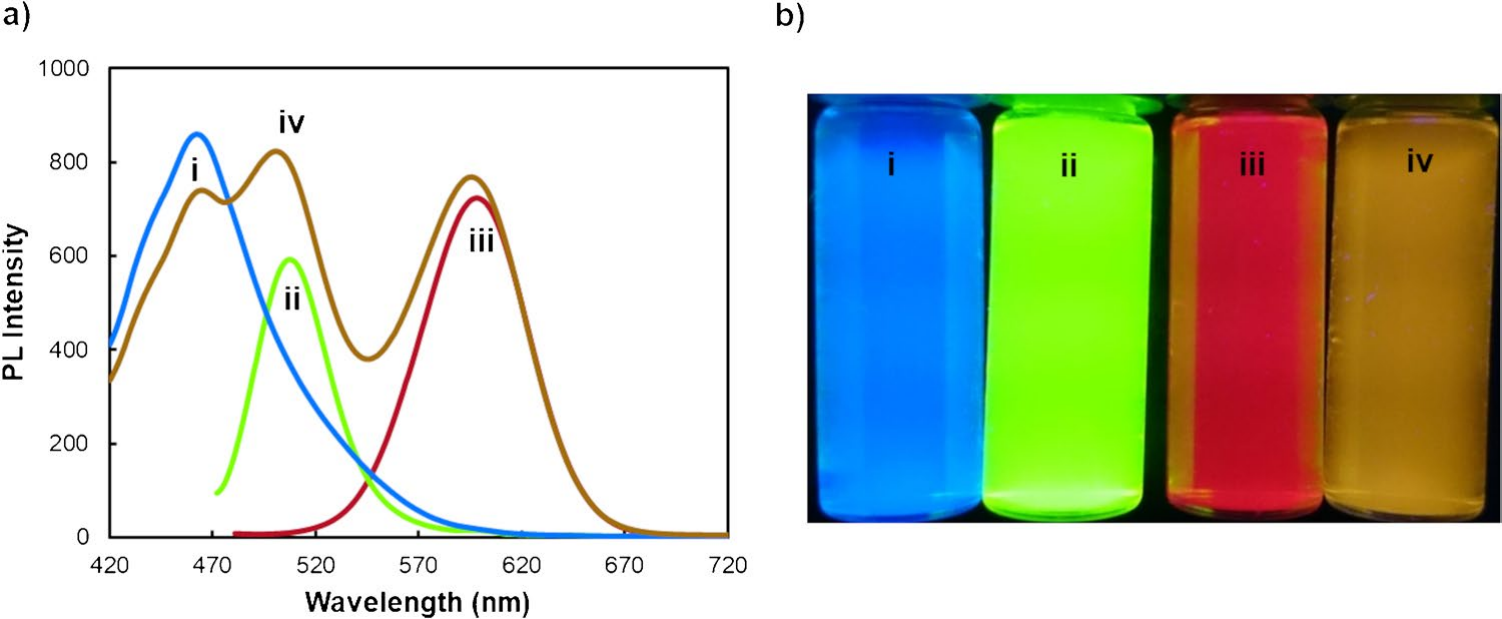
**a** PL spectra and **b** fluorescence images (under a 365-nm UV lamp) of CDs (i), GSH-CdTe QDs (ii), MPA-CdTe QDs (iii), and combined nanoprobe (iv)

All the synthesized QDs underwent characterization using both steady-state and time-resolved fluorimetry. All nanomaterials displayed nearly symmetric and narrow emission bands, with a maximum emission peak at 462, 508, and 598 nm for CDs, GSH-CdTe, and MPA-CdTe, respectively (Fig. 1 and Table 1). Furthermore, the PL lifetimes for these QDs were measured as 8.17 ± 0.02, 58.5 ± 0.4, and 54 ± 3 ns for CDs, GSH-CdTe, and MPA-CdTe, respectively (Fig. S1a and Table S1—supplementary material).

The data obtained from time-resolved PL measurements (Fig. S1b—supplementary material) of the different nanoparticles indicated that the PL decay could be appropriately modelled using two or three exponential decay kinetics. As anticipated, the composition (carbon-based and semiconducting QDs) of the nanoparticles and the nature of the surface capping ligand play a preponderant role in determining the PL lifetime values. Indeed, the PL lifetimes of binary CdTe QDs were roughly six times longer than those observed with CDs.

Additionally, the passivation of CdTe QDs using GSH resulted in shorter PL lifetimes compared to nanocrystals capped with MPA. This difference can be attributed to the distinct chemical nature and binding affinities of these ligands to the surface of the QDs. The passivation effect of capping ligands is crucial in determining the optical properties of QDs. Ligands like GSH and MPA not only stabilize the QDs but also play a significant role in passivating surface defects that can act as non-radiative recombination centres. MPA, with its strong binding affinity to the QD surface, provides more effective passivation of surface states, thereby reducing the non-radiative recombination processes and resulting in longer PL lifetimes. These results suggest that the molecular structure and binding characteristics of the capping ligands play a critical role in determining the electronic environment at the QD surface, which in turn influences the PL dynamics.

### Optimization of CDs/GSH-CdTe/MPA-CdTe sensing platform/development of a triple-emission PL probe

The concentrations of all synthesized quantum dots were standardized to ensure uniform fluorescence intensity, as outlined in subsection “Fluorometric measurement procedure”. Consequently, the final concentrations of GSH- and MPA-CdTe QDs were 2.8 and 9.2 µg mL^−1^ (w/v), respectively, while for CDs, a dilution factor of 1:25,000 from the crude solution was used.

The mixture of these three QD probes resulted in three well-defined emission peaks (Fig. 1a). When comparing the combined spectra profile with the individual spectra of each QD, a slight hypsochromic shift of the maximum emission wavelength was observed for GSH-CdTe QDs (from 508 to 500 nm), while the maximum emission wavelength remained practically unaffected for CDs and MPA-CdTe QDs. Additionally, in the combined nanoprobe, the PL emission intensity of CDs was slightly inhibited, whereas the PL emission intensity of GSH-CdTe exhibited an increase compared to each nanomaterial alone. These modifications in the PL intensity can be attributed to the overlap of the emission spectra of CDs and GSH-CdTe, leading to an increase in the PL intensity of the latter. An inner filter effect between both nanoparticles can also contribute to these changes.

**Fig. 2:**
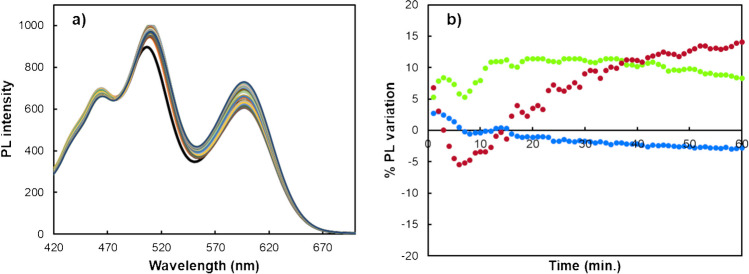
**a** PL spectra of the triple-emitting QDs-based nanoprobe during 60 min. **b** PL emission intensity evolution throughout 60 min for CDs (blue circle), GSH-CdTe (green circle), and MPA-CdTe (red circle) QDs

The stability of the probe solutions plays a crucial role in influencing the reliability and precision of consecutive fluorometric measurements performed during sample analysis. Therefore, a comprehensive investigation into the aqueous solution stability of the PL intensity of the combined nanoprobe was undertaken. Considering this, the fluorescence intensity of the sensing platform was evaluated for 60 min (Fig. 2). It was observed that, while the fluorescence intensity of the carbon dots (CDs) remained relatively stable, the fluorescence intensity of both binary QDs exhibited notable variations. Therefore, the nanohybrid probe mixture was prepared immediately before the acquisition of fluorescence spectra to mitigate any potential impact of probe instability on the obtained results.

To further ensure consistency during measurements, blank measurements were conducted alongside each analysis set. This approach provided a means to account for any background variations in PL intensity, confirming that observed fluorescence changes could be specifically attributed to interactions with metal ions rather than random fluctuations within the probe itself. By freshly preparing the nanohybrid probe mixture and incorporating blank controls, stable and reproducible results were ensured across all measurements.

### Assessment of triple-emitting quantum dots probe reactivity towards diverse metal ions

#### Reactivity of the combined nanoprobe

**Fig. 3:**
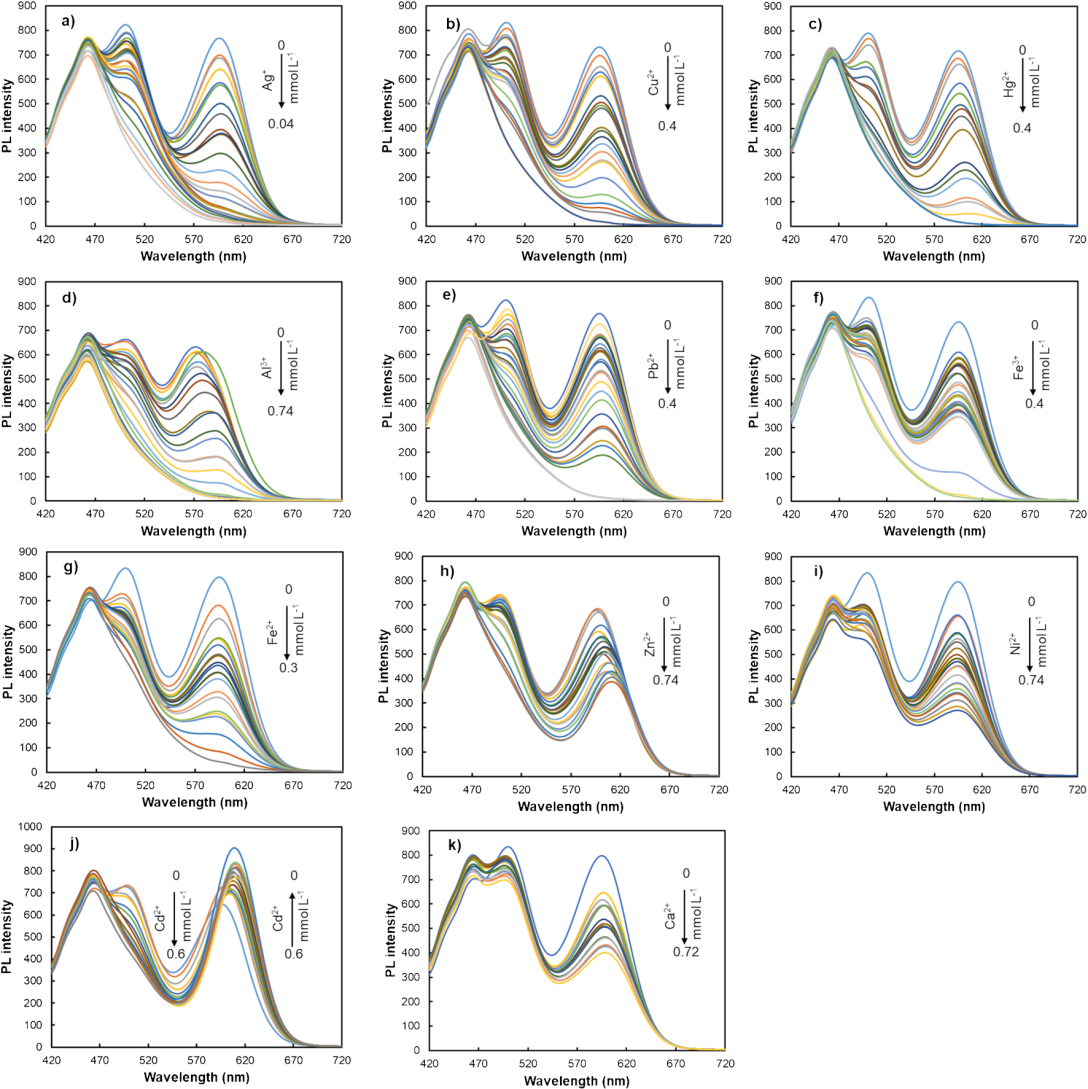
PL spectra of the triple-emitting QDs-based nanoprobe upon interaction with different metal ions such as **a** Ag^+^, **b** Cu^2+^, **c** Hg^2+^, **d** Al^3+^, **e** Pb^2+^, **f** Fe^3+^, **g** Fe^2+^, **h** Zn^2+^, **i** Ni^2+^, **j** Cd^2+^, and **k** Ca^2+^

To evaluate the reactivity of the combined probe, the interaction of the triple-emitting QD probe with increasing concentrations of various metals (namely Ag^+^, Cu^2+^, Hg^2+^, Al^3+^, Pb^2+^, Fe^3+^, Fe^2+^, Zn^2+^, Ni^2+^, Cd^2+^, and Ca^2+^) was tested. The results depicted in Fig. 3 revealed that, while the PL emission of both GSH and MPA-capped binary QDs was quenched by all metal ions, the extent of this effect varied. Additionally, this quenching exhibited concentration dependence, with a noticeable increase at higher analyte concentrations. An exception to this effect was found in the case of the interaction between cadmium and MPA-CdTe QDs, where the PL intensity increased with the concentration of the metal.

The pH of the QD probe solution was measured in the absence and presence of increasing concentrations of metal ions. No significant pH variations (from 6.9 to 6.5) were observed, indicating that the decrease in PL intensity is not attributed to pH changes but rather to the specific interaction between QDs and metal ions. This suggests that the method is effectively pH-independent under the tested conditions.

The observed changes in PL intensity provide valuable insights into the underlying reaction mechanisms. The fluorescence enhancement upon adding Cd^2^⁺ can primarily be attributed to the passivation of surface defects in the CdTe QDs. This passivation reduces non-radiative recombination pathways, promoting radiative recombination and consequently leading to an increase in PL intensity [20, 49]. The quenching of PL intensity for most metal ions can be attributed to photo-induced electron transfer (PET). In this process, metal ions act as quenchers by facilitating PET, where excited electrons from the QDs are transferred to the metal ions, resulting in reduced PL emission. This disrupts the electronic transitions within the QDs, leading to a decrease in emission intensity [2, 21, 24].

In addition to PL intensity changes, deviations in the maximum emission wavelength were also observed in some cases, particularly in the emission band corresponding to the MPA-CdTe QDs. Examples of this include the bathochromic shift upon the interaction with mercury (7 nm), zinc (13 nm), aluminium (21 nm), and cadmium (14 nm).

This phenomenon, characterized by a shift in the maximum emission towards longer wavelengths, can be attributed to different mechanisms. These include variations in surface charge or ligands of the QDs, as well as the adsorption of metals on the QDs’ surface leading to an increase in size, and the formation of trap radiative recombination centres [20, 21, 24]. Modifications in surface charge or ligands induced by the interaction with metal analytes play a crucial role in promoting the redshift by altering electrostatic repulsions between nanoparticles and fostering their aggregation. This aggregation increases particle size and contributes to the observed redshift. Similarly, this mechanism leads to PL quenching due to its adverse effects on nanoparticle dispersion stability in aqueous media. Another possible explanation for the redshift involves the incorporation of metal ions into the QDs’ surface, forming a metal–chalcogen structure. This increases particle size, which in turn reduces the band gap. Additionally, the incorporation of impurities into the nanocrystal structure can introduce intermediate energy states, such as trap states between the valence and conduction bands. These mid-gap states influence the relaxation dynamics of QDs, potentially enabling charge carrier transfer within trap radiative recombination centres [20, 21, 24].

On the other hand, the PL intensity of the CDs remained practically unchanged after the addition of increasing metal ion concentrations, except for slight quenching effect observed with Fe^3+^, Al^3+^, and Pb^2+^.

The results indicate that, in contrast to the PL data obtained using single-emitter QDs, the combined nanoprobe enables the acquisition of a specific analyte-response profile for each tested metal ion. Indeed, the use of multi-emitter nanoprobes enhances selectivity for target analytes. Traditional PL methodologies, relying on one-directional signal changes, are susceptible to interference. However, employing three different QDs with distinct affinities for specific analytes allows multi-point detection. This approach provides supplementary and more informative data for analyte determination, reducing the potential occurrence of signal interferents.

In fact, the high reactivity of QDs coupled with their exceptional optical and electronic properties makes them essential in the development of PL analytical methodologies. However, one of the limitations of PL-based sensing with QDs, often highlighted in the literature, is their limited selectivity. To overcome this, various strategies have been proposed, with chemometric data analysis emerging as one of the most promising. With this in mind, the obtained data were analysed using chemometric models to evaluate the ability of the combined nanoprobe to discriminate and quantify each tested metal ion.

#### Chemometric analysis towards individual metal ions samples

As previously discussed in subsection “Chemometric models for data analysis,” different chemometric models were assayed. In this subsection, only samples containing a single metal ion were analysed. Therefore, PCA was applied to datasets containing only one metal ion individually. In this context, the outliers spotted in each dataset were removed before applying PLS and PLS-DA. PCA was also used to verify the formation of clusters (Fig. S2—supplementary material).

Through the analysis of the score map of principal component 1 against principal component 2, the formation of the cluster is visible, as observed with Ag^+^, Cd^2+^, and Al^3+^. This is particularly evident for higher concentrations of each metal, where the scores tend towards regions outside the centre of the score map. This observation suggests the application of supervised models, namely PLS for quantification and PLS-DA for discrimination purposes.

##### Quantification through PLS

After removing outliers, the PLS-1 models were developed using PL data samples that contained only one metal ion, as previously discussed. Most of the outliers that were removed were from samples that exhibited total quenching of the GSH-CdTe and MPA-CdTe capped binary QDs. Table 1 and Fig. S3 (available in supplementary material) present the calibration and validation results of all PLS models, considering all spectral regions simultaneously. Details regarding the optimization of the PLS models can be found in the supplementary material, Table S2. The PLS models were validated using all spectral regions together (the spectral emission of all QDs), as they generally provided the best results and were not statistically different from other PLS models that considered different spectral regions and showed better results in some parameters (e.g., RMSEC, RMSECV, R^2^_C_, and R^2^_CV_). Moreover, considering the complexity of further experiments, we believe this is the best option for samples containing mixtures of metal ions.

The results demonstrate the suitability of the developed PL probe for the accurate quantification of these metal ions. In fact, ten out of eleven PLS models exhibited R^2^_P_ values higher than 0.9, indicating excellent model performance [50]. Moreover, seven out of eleven PLS models (Ag^+^, Cu^2+^, Hg^2+^, Al^3+^, Pb^2+^, Fe^3+^, Fe^2+^) achieved concentration values in the nmol L^−1^ range.

**Table 1:** Calibration and validation results for the PLS models after mean centring of PL data

	Ag^+^	Al^3+^	Ca^2+^	Cd^2+^	Cu^2+^	Fe^2+^	Fe^3+^	Hg^2+^	Ni^2+^	Pb^2+^	Zn^2+^
	Calibration										
Spectral region	R1 + R2 + R3										
Range^a^	Up to 1.00E-03	Up to 6.00E-02	Up to 7.20E-01	Up to 2.00E-01	Up to 7.00E-04	Up to 6.00E-02	Up to 7.40E-03	Up to 3.20E-04	Up to 7.40E-01	Up to 4.00E-04	Up to 7.40E-01
LV	2	4	3	3	1	4	3	5	5	3	4
RMSEC^a^	5.74E-05	8.29E-03	0.040	1.66E-03	3.45E-05	4.04E-03	3.93E-04	9.43E-06	1.21E-02	1.37E-05	2.27E-02
RMSECV^a^	7.29E-05	2.48E-02	0.072	0.0102	4.33E-05	6.90E-03	6.71E-04	1.88E-05	5.56E-02	2.16E-05	4.98E-02
R^2^_C_	0.935	0.992	0.971	0.999	0.969	0.957	0.978	0.991	0.997	0.986	0.984
R^2^_CV_	0.897	0.937	0.907	0.982	0.954	0.894	0.935	0.966	0.938	0.966	0.925
	Validation										
RMSEP^a^	6.28E-05	1.01E-02	0.0446	6.88E-03	4.09E-05	6.72E-03	8.03E-04	1.60E-05	4.96E-02	1.46E-05	3.15E-02
R^2^_P_	0.931	0.977	0.957	0.988	0.971	0.887	0.922	0.970	0.958	0.990	0.978

^a^Values expressed in mmol L^−1^

##### Discrimination through PLS-DA

The goal of discrimination is to distinguish between the different metal ions that exhibit overlapping or similar PL responses, as these metal ions often coexist in complex matrices such as environmental samples and industrial effluents. The discrimination of eleven metal ions using QD probes has never been accomplished due to their reactivity upon interaction with QDs. In this sub-section, our objective was to demonstrate the suitability of the triple-emitting QD probe for the accurate discrimination of several ions. The PLS1-DA models were constructed using PL data samples with just one metal ion, after removing outliers, as mentioned previously. Additionally, in the PLS-DA models, the blank signals were also removed. The study of the different spectral regions aimed to demonstrate that using the entire signal (R1 + R2 + R3) allows for better discrimination than using only a single-emitting QD probe (such as R1, R2, or R3 individually).

The best results were obtained (Table 2) when using the spectral combination of R2 + R3 (GSH-CdTe and MPA-CdTe capped binary QDs, respectively) and the entire spectral region (CDs and GSH-CdTe and MPA-CdTe capped binary QDs, respectively). It is noticeable that when using only one QD, the percentage of correct predictions is not as high. However, when the QDs are combined, the percentage of correct predictions increases significantly. It is also evident that the GSH-CdTe and MPA-CdTe capped binary QDs were the most effective for discriminating these metal ions. In fact, the combination of these QDs allowed for around 90% correct predictions. The use of the tripe-emission nanoprobe (considering all spectral regions together) also enabled around 90% of correct predictions, attesting to the suitability of the developed methodology. Given the complexity of the subsequent experiments, we remain confident that this is the optimal choice for samples containing mixtures of metal ions.

**Table 2:** Calibration and validation results for the PLS-DA models using 9 LVs, with prior mean centring of PL data

	Spectral regions						
	R1	R2	R3	R1 + R2	R1 + R3	R2 + R3	R1 + R2 + R3
	Calibration						
Percentage of correct predictions	48.8	76.7	67.4	83.7	83.7	93.0	93.0
	Validation						
Percentage of correct predictions	57.1	54.0	73.0	84.1	84.1	90.5	88.9

To understand which metal ions were more accurately discriminated, the obtained confusion matrix using the entire spectral region is provided in Table 3. The additional confusion matrices obtained when using the other spectral regions are included in the supplementary material (Tables S3-S8).

**Table 3:** Confusion matrix for PLS-DA metal ions discrimination model with the entire spectral regions (R1 + R2 + R3), 9 LVs, and mean-centred PL data for validation

Real metals ions	Predicted metal ions										
	Ag^+^	Ca^2+^	Cd^2+^	Cu^2+^	Fe^2+^	Fe^3+^	Hg^2+^	Ni^2+^	Pb^2+^	Zn^2+^	Al^3+^
Ag^+^	$\frac{5}{6}$	0	0	0	0	$\frac{1}{6}$	0	0	0	0	0
Ca^2+^	0	$\frac{4}{5}$	0	0	0	0	0	$\frac{1}{5}$	0	0	0
Cd^2+^	0	0	$\frac{3}{3}$	0	0	0	0	0	0	0	0
Cu^2+^	0	0	0	$\frac{3}{5}$	0	$\frac{1}{5}$	0	0	0	0	$\frac{1}{5}$
Fe^2+^	0	0	0	0	$\frac{6}{6}$	0	0	0	0	0	0
Fe^3+^	0	0	0	0	0	$\frac{8}{8}$	0	0	0	0	0
Hg^2+^	0	0	0	$\frac{1}{5}$	0	0	$\frac{3}{5}$	0	$\frac{1}{5}$	0	0
Ni^2+^	0	0	0	0	0	0	0	$\frac{7}{7}$	0	0	0
Pb^2+^	0	0	0	0	0	0	0	0	$\frac{7}{7}$	0	0
Zn^2+^	0	0	0	0	0	$\frac{1}{6}$	0	0	0	$\frac{5}{6}$	0
Al^3+^	0	0	0	0	0	0	0	0	0	0	$\frac{5}{5}$

The analysis of the confusion matrix reveals that six metal ions, namely Cd^2+^, Fe^2+^, Fe^3+^, Ni^2+^, Pb^2+^, and Al^3+^, were consistently predicted accurately (100% of correct predictions). The poorest predictions occurred for Cu^2+^ and Hg^2+^, with only 60% of the validation samples being correctly predicted. Misclassifications with Cu^2+^ occurred with Fe^3+^ and Al^3+^, while for Hg^2+^, misclassifications occurred with Cu^2+^ and Pb^2+^. The remaining metal ions, namely Ag^+^, Ca^2+^, and Zn^2+^, showed correct predictions of around 80%. These results attest to the suitability of the developed methodology, based on a triple emission nanoprobe, for discriminating metal ions.

### Multiplexed detection of metal ions using the combined nanoprobe

#### Kinetic behaviour of the interaction of the nanoprobe and each metal ion

**Fig. 4:**
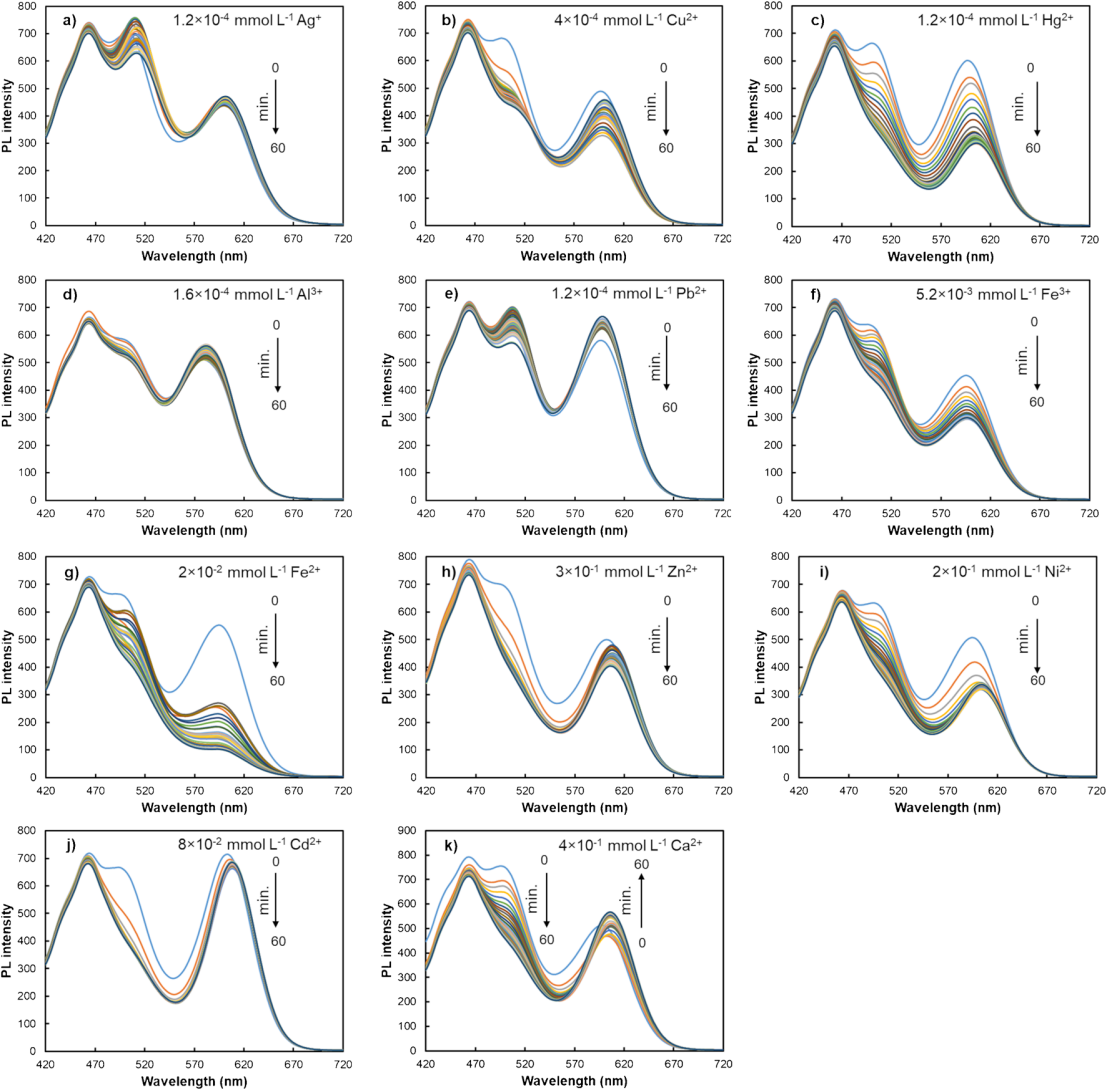
PL spectra evolution (60 min) of the triple-emitting QDs-based nanoprobe upon interacting with various metal ions: **a** Ag^+^, **b** Cu^2+^, **c** Hg^2+^, **d** Al^3+^, **e** Pb^2+^, **f** Fe^3+^, **g** Fe^2+^, **h** Zn^2+^, **i** Ni^2+^, **j** Cd^2+^, and **k** Ca^2+^

The previously obtained results demonstrate the efficiency of the combined probe for individually quantifying and discriminating each metal in solution. However, the main objective of this work was to detect and/or discriminate two or more metal ions simultaneously in a mixture through a single measurement. To achieve this goal, the kinetic behaviour of the combined probe’s interaction with each metal, individually, was assessed over a period of 60 min (Fig. 4).

By enhancing the complexity of the data collected during each sample analysis, one can anticipate obtaining more finely resolved quantitative or qualitative analytical estimations, thereby achieving higher selectivity. Therefore, to explore the possibility of using kinetic data to enhance analytical estimations in multiplexed determinations, the behaviour of the triple-emitting QDs-based nanoprobe was assessed with each metal individually. The concentration used for each metal was chosen seeking to enable the observation of the signals corresponding to the three nanomaterials present in the mixture at the beginning of the measurements.

It is important to emphasize that the evaluation of the optical properties of the triple-emission probe in the presence of metal ions was always conducted with reference to the probe’s behaviour in the absence of ions (blank). This approach ensures measurement reliability, as each fluorescence reading in the presence of metal ions was evaluated by comparing it with the corresponding blank sample. Furthermore, to account for potential fluctuations in the probe, each probe mixture was prepared immediately before acquiring the fluorescence spectra, with a blank measurement consistently included alongside each sample set. These steps ensure that potential probe instabilities are accounted for in each analysis, allowing for accurate and reliable comparative evaluation of the ion interactions.

After analysing the changes in PL spectra resulting from the interaction between the combined nanoprobe and each metal (Fig. 4), it is evident that certain metals such as Cu^2+^, Hg^2+^, Fe^3+^, Fe^2+^, and Ni^2+^ induce significant alterations in the combined probe over time. On the contrary, certain metal ions, like Ag^+^ and Al^3+^, have a minimal impact. Notably, even in cases where substantial changes in the optical properties of the QDs occur over time, these alterations are noticeable within the first few minutes of interaction and remain practically unchanged for longer periods. As a result, to achieve a balance between acquiring more complex data, identifying moments of pronounced signal changes, and avoiding excessively prolonged readings, the analysis of PL data focused on the first 5 min of interactions between the metal ions and the PL nanoprobe in future assays.

#### Quantification through PLS (first-order data) and U-PLS (second-order data)

Three different binary metal combinations were selected to assess the suitability of the developed nanoprobe, namely Pb^2+^ with Hg^2+^, Al^3+^ with Hg^2+^, and Ag^+^ with Hg^2+^. Both first-order and second-order data were employed to determine whether there was a significant improvement in the accuracy of the models.

##### Binary mixture with Pb^2+^ and Hg^2+^

The first combination of two metal ions tested was Pb^2+^ with Hg^2+^. Several experimental sets of mixtures of these two metal ions were prepared due to some unexpected behaviours, notably related to the molar ratio of the concentrations of both metal ions, which will be further discussed. In the first experiment, a total of 22 solutions were prepared with concentration ranges of up to 1.02 × 10^−4^ mmol L^−1^ for Pb^2+^ and up to 8.13 × 10^−5^ mmol L^−1^ for Hg^2+^. From these solutions, 15 were used for calibration and the remaining 7 solutions were used for validation, according to a dataset division of 70% for calibration and 30% for validation. The results demonstrated that using second-order data (PL data during 5 min) instead of first-order data (PL data at time zero) resulted in better accuracy for the developed models in terms of all analysed parameters, namely RMSECV, RMSEP, R^2^_CV_, and R^2^_P_ (Table S9). Moreover, the PLS and U-PLS models for Pb^2+^ determination were not as accurate as the standards with the lowest concentration were poorly predicted. Therefore, it was decided to increase the concentration range of Pb^2+^ for the second experiment.

**Fig. 5:**
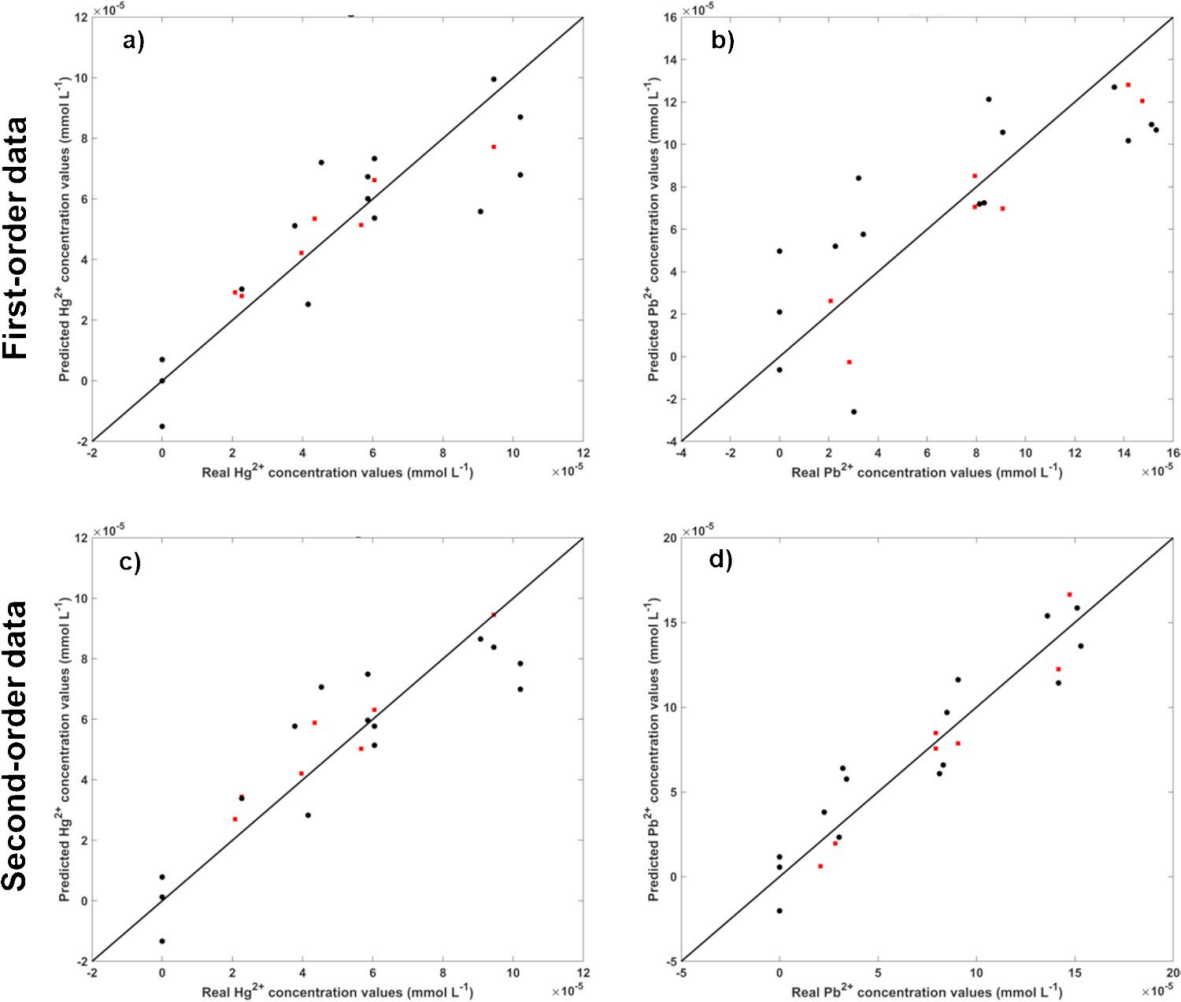
Real values versus the predicted cross-validation (black circle) and validation (red square) values obtained for PLS (**a** and **b**) and U-PLS (**c** and **d**) models considering the entire spectral range and the mixture of Hg^2+^ (**a** and **c**) and Pb^2+^ (**b** and **d**). Note that PL data was mean-centred previously

In the second experiment, a total of 22 solutions were prepared with concentration ranges of up to 1.53 × 10^−4^ mmol L^−1^ for Pb^2+^ and up to 8.13 × 10^−5^ mmol L^−1^ for Hg^2+^. As expected, the accuracy for Pb^2+^ determination increased, but unexpectedly, the accuracy for Hg^2+^ decreased. Due to this unexpected result, another experiment was planned. It was decided to adjust the molar ratio between Pb^2+^ and Hg^2+^ to half of that in the first two experiments, specifically 1.5 (whereas in the first and second experiments, the molar ratios considering the highest concentration solutions were 1.25 and 1.88, respectively). In the third experiment, 22 solutions were prepared with concentration ranges of Pb^2+^ up to 1.53 × 10^−4^ mmol L^−1^ and Hg^2+^ up to 1.02 × 10^−4^ mmol L^−1^. The results obtained for both PLS and U-PLS models were accurate, with the second-order data (U-PLS model) showing particularly high accuracy, with R^2^_P_ values higher than 0.96 for both parameters (Fig. 5).

This demonstrates the reliability and accuracy of the models, as R^2^_P_ values higher than 0.9 indicate excellent models [50]. Moreover, this is the first reported instance where the molar ratio concentrations of the metal ions significantly contribute to the accuracy of the respective PLS and U-PLS models.

##### Binary mixture with Al^3+^ and Hg^2+^

**Fig. 6:**
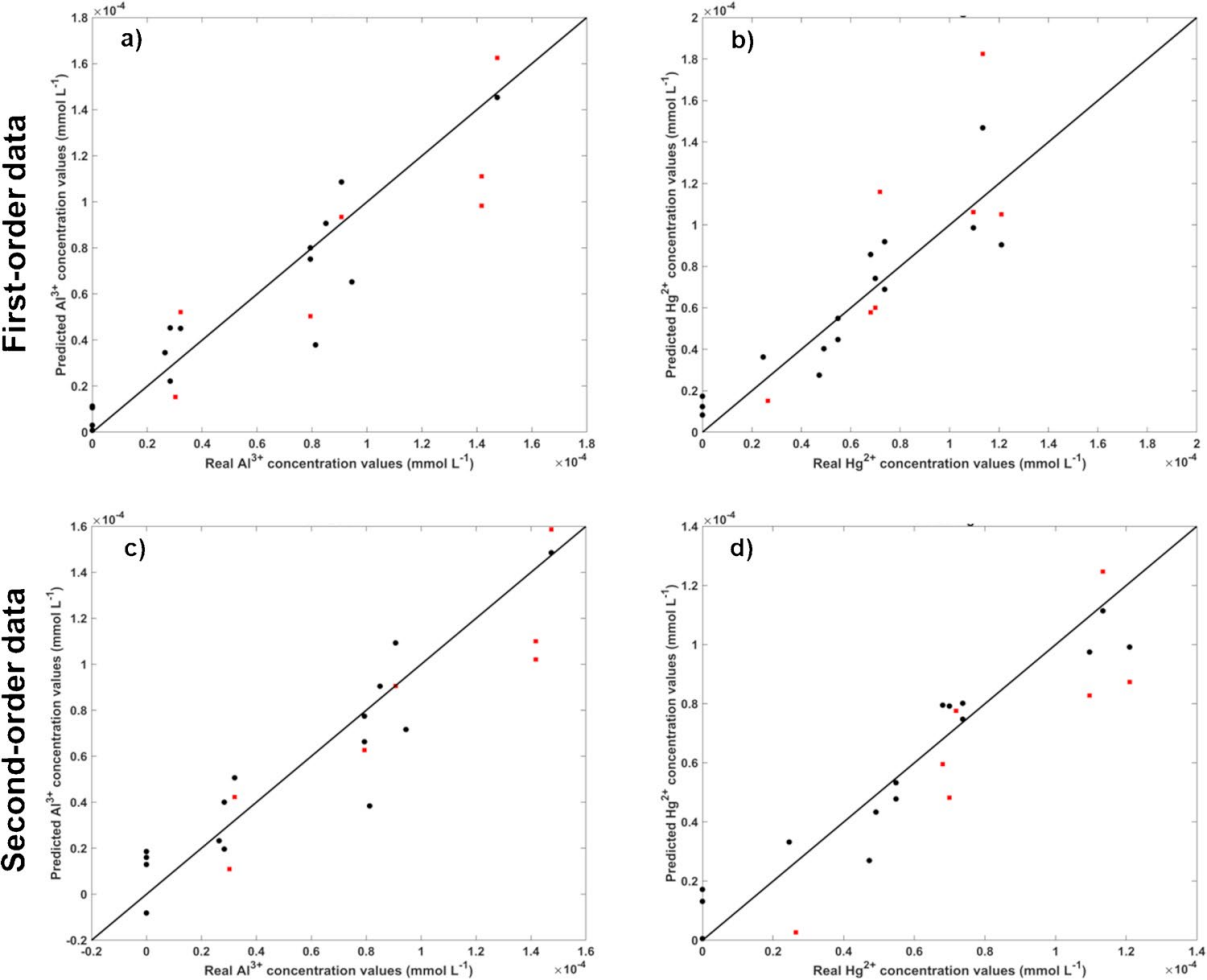
Real values versus the predicted cross-validation (black circle) and validation (red square) values obtained for PLS (**a** and **b**) and U-PLS (**c** and **d**) models considering the entire spectral range and the mixture of Al^3+^ (**a** and **c**) and Hg^2+^ (**b** and **d**). Note that PL data was mean-centred previously

The second combination tested involved Al^3+^ with Hg^2+^ metal ions. Initially, 22 solutions were prepared in the first experiment, with concentration ranges of Al^3+^ up to 1.53 × 10^−4^ mmol L^−1^ and Hg^2+^ up to 8.13 × 10^−5^ mmol L^−1^. However, the results (Table S10—Supplementary Material) were not as accurate as expected for either metal ions. Additionally, there was no significant difference between the results obtained from first- and second-order data analysis. Given the insights into the influence of the molar concentrations between metal ions, it was decided to conduct further experiments to adjust the molar ratio concentrations of Al^3+^ and Hg^2+^. Initially, the molar ratio considering the highest concentration solutions of Al^3+^ and Hg^2+^ metal ions was 1.88. Therefore, in the second experiment, a new set of solutions were prepared with a molar ratio of 1.5. In this experiment, 22 solutions were prepared with concentration ranges of Al^3+^ up to 1.53 × 10^−4^ mmol L^−1^ and Hg^2+^ up to 1.02 × 10^−4^ mmol L^−1^. Unfortunately, the results for Hg^2+^ metal ion determination were worse than those obtained in the first experiment. Subsequently, it was decided to decrease the molar ratio of Al^3+^ and Hg^2+^ metal ions aiming to further improve the results of Hg^2+^ metal ion determination. Therefore, in the third experiment, a total of 22 solutions were prepared with concentration ranges of Al^3+^ and Hg^2+^ ions up to 1.47 × 10^−4^ mmol L^−1^ and 1.21 × 10^−4^ mmol L^−1^, respectively, resulting in a molar ratio concentration of 1.22. In this case, the accuracy of both PLS (first-order data) and U-PLS (second-order data) models improved for both metal ions (Table S10—supplementary material and Fig. 6). This improvement was particularly noticeable when considering the second-order data (U-PLS), aligning with the results obtained for Hg^2+^ and Pb^2+^ metal ions mixtures.

Once again, the results demonstrate the accuracy and reliability of the developed methodology for determining mixtures of metal ions, especially evident when using second-order data with R^2^_P_ around 0.90, indicating excellent models [50]. Furthermore, the significant influence of the molar ratio on the accuracy of the respective PLS and U-PLS models was evident once more.

The influence of molar ratio concentrations underscores a previously neglected aspect, suggesting the necessity for further investigation. This likely stems from a competitive mechanism in which various factors such as ionic radius and metal affinity, among others, may influence outcomes. Further research is needed to elucidate this complex interplay and its implications thoroughly.

##### Binary mixture Ag^+^ and Hg^2+^

Lastly, Ag^+^ and Hg^2+^ were combined in a set of several mixtures, namely 22 solutions. The concentration ranges were up to 1.02 × 10^−4^ mmol L^−1^ and 8.13 × 10^−5^ mmol L^−1^, for Ag^+^ and Hg^2+^, respectively. In this case, only one set of experiments was needed to attain good results, due to the acquired knowledge in terms of molar ratio from prior experiments. The results obtained when using first- or second-order data were very similar (Table S11 and Fig. S4—supplementary material).

In fact, both types of data revealed a high accuracy for the quantification of both metal ions in the prepared mixtures, with R^2^_P_ around 0.90 which indicates excellent models [50].

##### Ternary mixture Ag^+^, Hg^2+^, and Pb^2+^

**Fig. 7:**
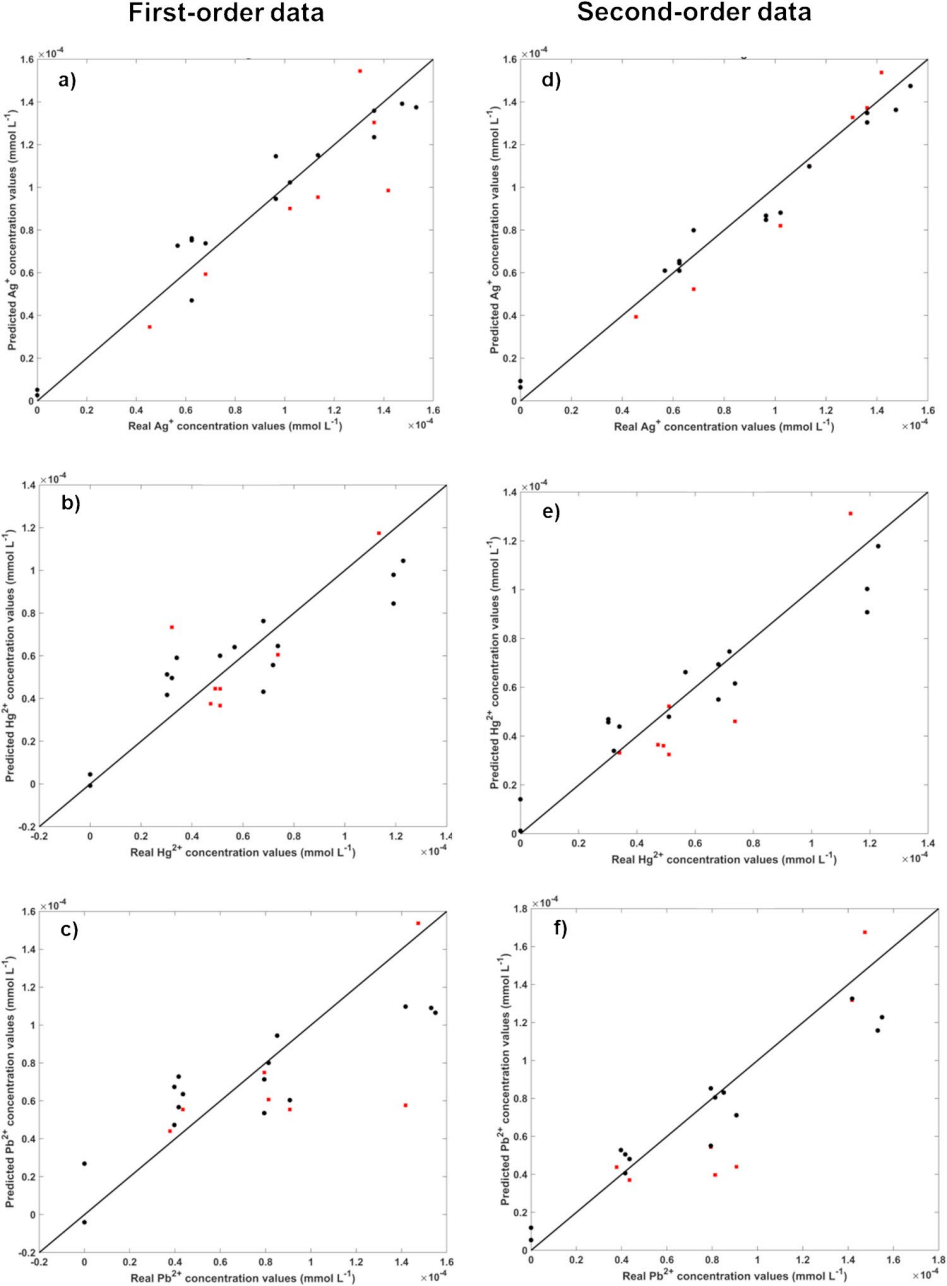
Real values versus the predicted cross-validation (black circle) and validation (red square) values obtained for PLS (**a**, **b**, and **c**) and U-PLS (**d**, **e**, and **f**) models considering the entire spectral range and the ternary mixture of Ag^+^ (**a** and **d**), Hg^2+^ (**b** and **e**), and Pb^2+^ (**c** and **f**). Note that PL data was mean-centred previously

Taking into account the suitability of the developed nanoprobe for accurately determining various binary metal mixtures, a ternary metal combination containing Ag^+^, Hg^2+^, and Pb^2+^ was also prepared. Once again, first-order and second-order data were employed to determine whether there was a significant improvement in the accuracy of the models. An experimental set was prepared, consisting of 22 solutions with concentration ranges of Ag^+^, Hg^2+^, and Pb^2+^ up to 1.53 × 10^−4^ mmol L^−1^, 1.23 × 10^−4^ mmol L^−1^, and 1.55 × 10^−4^ mmol L^−1^, respectively. The choice of concentrations was based on observations made in the previous binary mixtures. Concentration ranges that provided good discrimination between the metal ions were used, ensuring that each ion generated distinct PL signals for robust analysis with second-order data. The obtained results are shown in Table 4 and Fig. 7.

**Table 4:** Calibration and validation results for the PLS (first-order data) and U-PLS (second-order data) models considering Ag^+^, Hg^2+^, and Pb^2+^ mixture. Note that the PL data were previously mean centred

	First-order data—PLS			Second-order data—U-PLS		
	Ag^+^	Hg^2+^	Pb^2+^	Ag^+^	Hg^2+^	Pb^2+^
LV	4	1	1	3	1	1
RMSECV^a^	1.07E-05	1.77E-05	2.61E-05	7.88E-06	1.27E-05	1.63E-05
R^2^_CV_	0.975	0.903	0.876	0.991	0.960	0.967
RMSEP^a^	2.12E-05	1.79E-05	3.58E-05	1.10E-05	1.56E-05	2.70E-05
R^2^_P_	0.872	0.759	0.648	0.981	0.926	0.891

^a^Values expressed in mmol L^−1^

Using second-order data (U-PLS models’ results with PL data during 5 min) resulted in significantly better results in terms of RMSECV, RMSEP, R^2^_CV_, and R^2^_P_ compared to using first-order data (PLS models’ results with PL data at time zero). This trend aligns with the results obtained in the binary mixtures.

The results obtained with U-PLS models were accurate for all the metals, with R^2^_P_ values higher than 0.90 for Ag^+^ and Hg^2+^ and around 0.90 for Pb^2+^, indicating excellent models [50] and attesting to the suitability of the developed methodology. This is the first time ever reported of an accurate quantification of three metal ions simultaneously using this methodology. We believe this achievement was possible due to using second-order data and a triple-emitting nanoprobe, which allowed to observe the distinctive behaviours of these metal ions.

For mercury ion, maximum limits of detection (LOD) around 4.7 and 5.5 µg L^−1^ were obtained using second- and first-order data, respectively, while the World Health Organization (WHO) recommends a maximum limit of 6 µg L^−1^. For lead, maximum LOD around 5.2 and 7.9 µg L^−1^ were obtained for second- and first-order data, respectively, which is within the WHO-recommended maximum limit of 10 µg L^−1^. No WHO guideline has been established for silver ions. Therefore, the results obtained in this exploratory study are within the WHO recommendations for lead and mercury ions. Compared to the reference procedures, these figures of merit are inferior to the ones reported in the reference procedure. However, the proposed method is simpler, less labour-intensive, more cost-effective, and requires less maintenance. For instance, the Environmental Protection Agency (EPA) has established methods as reference procedures for the quantification of lead in drinking water, including inductively coupled plasma-mass spectrometry (ICP-MS), inductively coupled plasma-atomic emission spectrometry (ICP-AES), and graphite furnace atomic absorption spectroscopy (GFAAS), which yield LODs of 0.6, 42, and 0.7 µg L^−1^, respectively [51]. In terms of mercury ion detection, cold vapor atomic fluorescence spectrometry (CVAFS) is the EPA’s most sensitive standard method, with an LOD of 0.55 ng L^−1^ [52]. For silver ions, ICP-MS is among the EPA-recommended techniques, although no specific LOD is provided [53].

While the selectivity, sensitivity, and LOD of these reference procedures are superior to those of our developed methodology, our approach remains advantageous in terms of simplicity, reduced labour, and cost-effectiveness, particularly when considering maintenance costs compared to ICP-MS and CVAFS.

## Conclusion

Addressing the urgent global issue of metal ion pollution and its implications for human health and the environment, this study has presented a novel and efficient methodology for the simultaneous quantification of multiple metal ions. By employing a triple-emission nanoprobe integrating blue-emitting carbon dots and distinctly capped CdTe quantum dots, this research successfully demonstrated the capability for multiplexed detection of various metal ions.

The developed methodology exhibited remarkable accuracy by utilizing first- and second-order PL data and analysing it with advanced chemometric tools such as PCA, PLS regression, PLS-DA, and U-PLS regression models. Notably, the obtained *R*^2^ values for PLS and U-PLS surpassed 0.9 for several metal ions, even at concentrations lower than the mmol L^−1^ range, thus emphasizing the efficacy of the approach.

Furthermore, the study revealed the superiority of PL second-order data over PL first-order data, attributed to the distinctive time-based behaviour exhibited by the metal ions. Interestingly, it is the first time that the molar ratio of the metal ions tested is reported to have a significant impact on the accuracy of the developed models. Further studies are needed to understand the mechanism of competition among the metal ions using the developed nanoprobe.

The proposed triple-emission nanoprobe shows significant promise for practical applications in complex real samples. Future work will focus on adapting the nanoprobe for use in real samples by optimizing surface functionalization for enhanced selectivity and developing tailored sample preparation protocols to minimize matrix effects. Additionally, refining our chemometric models will be crucial to accurately analyse the complex data generated from real sample testing. These steps will ensure the method’s robustness and reliability for practical analytical applications.

## Supplementary Information

Below is the link to the electronic supplementary material.

Supplementary file1 (DOCX 1032 KB)
