# Supplementary material for: Chemometrically driven multiplexed metal ion detection using a triple emitting quantum dots–based nanoprobe
Source: Anal Bioanal Chem. 2024 Nov 26;417(2):417–33. doi: 10.1007/s00216-024-05661-7 (PMC11698780; doi:10.1007/s00216-024-05661-7)
Supplement: Supplementary file 1 — Supplementary file1 (DOCX 1032 KB) [file 216_2024_5661_MOESM1_ESM.docx]

**Supplementary material**

**Chemometrically-Driven Multiplexed Metal Ion Detection using a Triple Emitting Quantum Dots-Based Nanoprobe**

Rafael C. Castro, Ricardo N.M.J. Páscoa*, M. Lúcia M. F. S. Saraiva, João L.M. Santos*, David S.M. Ribeiro*


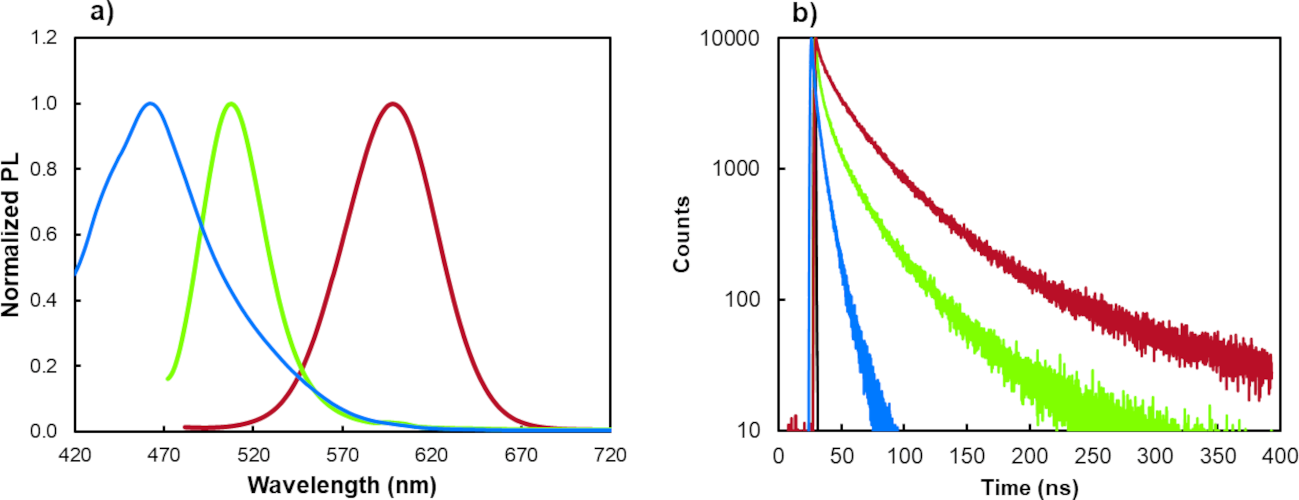


**Fig. S1.** **a)** Normalized PL spectra and **b)** PL decay curves of CDs (blue line), GSH-CdTe QDs (green line) and MPA-CdTe QDs.

**Table S1.** Maximum emission wavelength (λ_max_.), PL lifetime (τ**_average_**) and QY values of each synthesized nanomaterials.

| **QDs** | **λ_max._ (nm)** | **PL Lifetimes** | **1^st^ Decay Component** | **2^nd^ Decay Component** | **3^rd^ Decay Component** | **τ_average_ (ns)** | **QY (%)** |
| --- | --- | --- | --- | --- | --- | --- | --- |
| CDs | 462 | τ_i_ (ns) | 2.61 ± 0.02 | 9.33 ± 0.02 |  | 8.17 ± 0.02 | 19.9 ± 0.1 |
|  |  | B_i_ (%) | 42.7 | 57.3 |  |  |  |
| GSH-CdTe | 508 | τ_I_ (ns) | 17.3 ± 0.3 | 53.8 ± 0.3 | 3.13 ± 0.03 | 58.5 ± 0.4 | 47.3 ± 0.2 |
|  |  | B_i_ (%) | 49.2 | 31.5 | 19.3 |  |  |
| MPA-CdTe | 598 | τ_I_ (ns) | 27.0 ± 0.4 | 5.18 ± 0.09 | 74.2 ± 0.4 | 54 ± 3 | 62.3 ± 0.1 |
|  |  | B_i_ (%) | 53.5 | 7.3 | 39.3 |  |  |


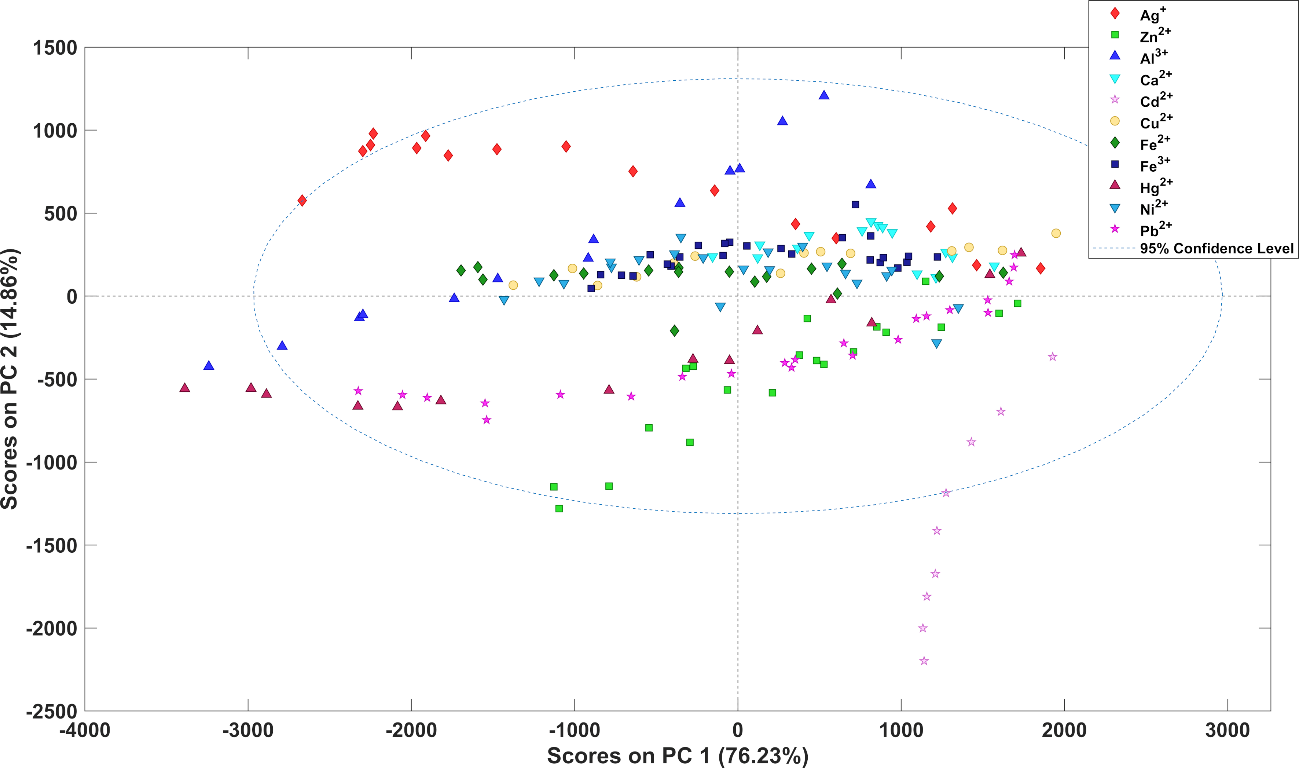


**Fig. S2.** Score plots obtained from PCA using the entire PL data after mean centering. The analysis reveals the distribution and clustering patterns of the data.


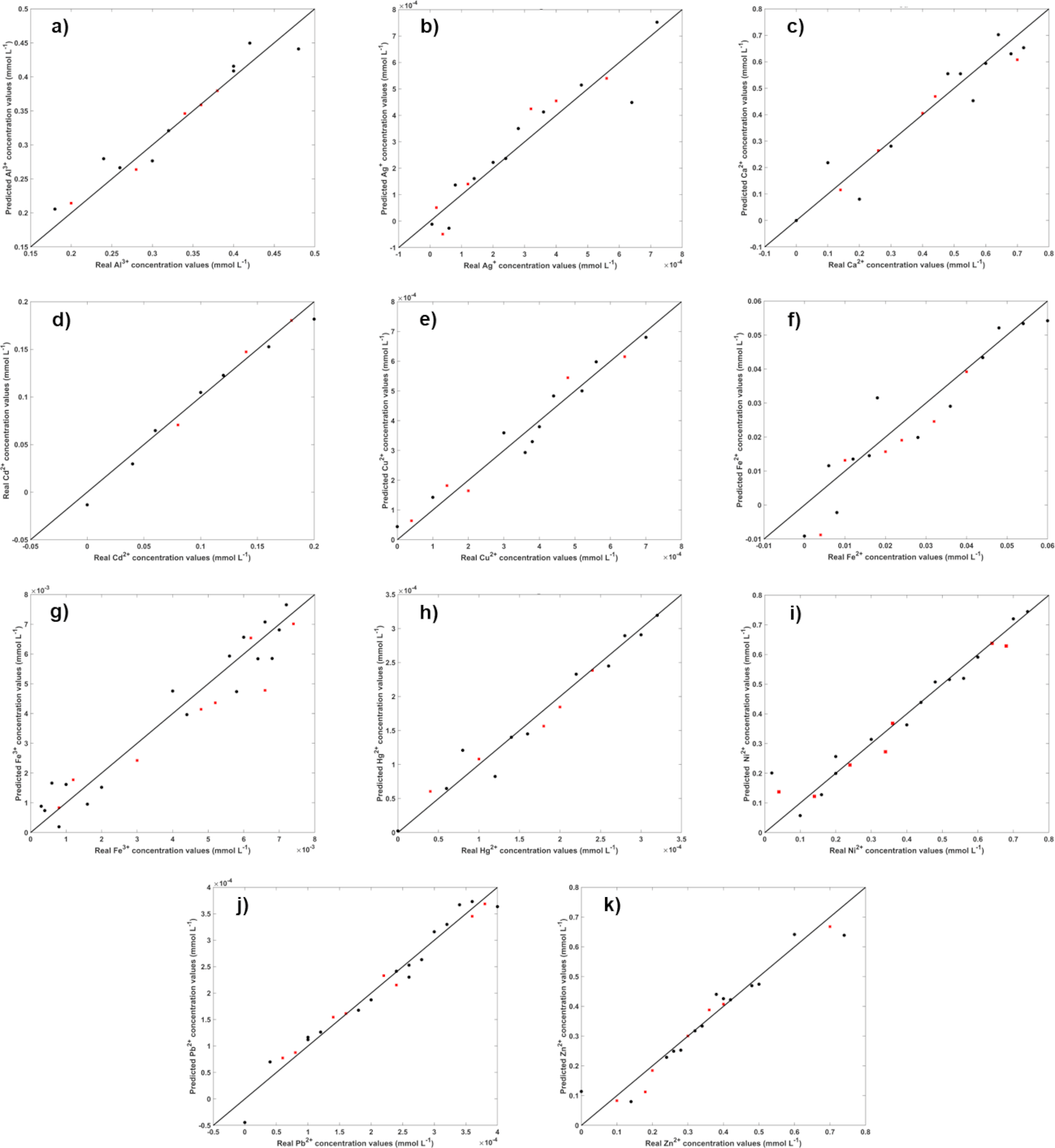


**Fig. S3**. Real values versus the predicted cross-validation (●) and validation (■) values obtained for all models considering the entire spectral range to **a)** Al^3+^, **b)** Ag^+^, **c)** Ca^2+^, **d)** Cd^2+^, **e)** Cu^2+^, **f)** Fe^2+^, **g)** Fe^3+^, **h)** Hg^2+^, **i)** Ni^2+^, **j)** Pb^2+^ and **k)** Zn^2+^. Note that PL data was mean-centered previously.

**Table S2.** Calibration results for all metals from optimized PLS models with prior mean centering of PL data.

|  | Spectral Regions | | | | | | |
| --- | --- | --- | --- | --- | --- | --- | --- |
|  | R1 | R2 | R3 | R1 + R2 | R1 + R3 | R2 + R3 | R1 + R2 +R3 |
|  | Ag^+^ | | | | | | |
| RMSEC | 9.00E-05 | 5.47E-05 | 5.98E-05 | 5.88E-05 | 5.61E-05 | 5.65E-05 | 5.74E-05 |
| RMSECV | 1.26E-04 | 7.47E-05 | 9.76E-05 | 7.92E-05 | 8.89E-05 | 7.14E-05 | 7.29E-05 |
| R^2^_C_ | 0.841 | 0.941 | 0.930 | 0.932 | 0.938 | 0.937 | 0.935 |
| R^2^_CV_ | 0.730 | 0.894 | 0.813 | 0.877 | 0.855 | 0.901 | 0.897 |
|  | Al^3+^ | | | | | | |
| RMSEC | 1.56E-02 | 8.74E-03 | 8.40E-03 | 9.83E-03 | 1.21E-02 | 8.22E-03 | 8.29E-03 |
| RMSECV | 5.98E-02 | 2.02E-02 | 9.70E-02 | 2.54E-02 | 4.06E-02 | 2.05E-02 | 2.48E-02 |
| R^2^_C_ | 0.971 | 0.991 | 0.992 | 0.989 | 0.983 | 0.992 | 0.992 |
| R^2^_CV_ | 0.721 | 0.952 | 0.626 | 0.931 | 0.826 | 0.956 | 0.937 |
|  | Ca^2+^ | | | | | | |
| RMSEC | 1.52E-01 | 4.76E-02 | 3.87E-02 | 6.03E-02 | 4.21E-02 | 4.11E-02 | 4.04E-02 |
| RMSECV | 2.59E-01 | 9.33E-02 | 5.91E-02 | 1.32E-01 | 6.73E-02 | 7.04E-02 | 7.25E-02 |
| R^2^_C_ | 0.582 | 0.959 | 0.973 | 0.934 | 0.968 | 0.970 | 0.971 |
| R^2^_CV_ | 0.102 | 0.845 | 0.937 | 0.694 | 0.919 | 0.912 | 0.907 |
|  | Cd^2+^ | | | | | | |
| RMSEC | 6.11E-03 | 1.03E-02 | 2.82E-03 | 1.04E-02 | 5.80E-03 | 1.88E-03 | 1.66E-03 |
| RMSECV | 3.07E-02 | 3.73E-02 | 3.07E-02 | 3.79E-02 | 3.09E-02 | 8.31E-03 | 1.02E-02 |
| R^2^_C_ | 0.991 | 0.975 | 0.998 | 0.974 | 0.992 | 0.999 | 0.999 |
| R^2^_CV_ | 0.794 | 0.680 | 0.904 | 0.672 | 0.844 | 0.990 | 0.982 |
|  | Cu^2+^ | | | | | | |
| RMSEC | 1.79E-04 | 4.24E-05 | 3.65E-05 | 4.08E-05 | 3.49E-05 | 3.59E-05 | 3.45E-05 |
| RMSECV | 5.80E-04 | 5.26E-05 | 4.24E-05 | 7.23E-05 | 4.39E-05 | 4.19E-05 | 4.33E-05 |
| R^2^_C_ | 0.169 | 0.954 | 0.966 | 0.957 | 0.969 | 0.967 | 0.969 |
| R^2^_CV_ | 0.292 | 0.929 | 0.954 | 0.888 | 0.953 | 0.955 | 0.954 |
|  | Fe^2+^ | | | | | | |
| RMSEC | 7.80E-03 | 4.75E-03 | 3.22E-03 | 2.47E-03 | 3.28E-03 | 4.35E-03 | 4.04E-03 |
| RMSECV | 3.23E-02 | 9.13E-03 | 8.26E-03 | 1.03E-02 | 5.15E-03 | 8.88E-03 | 6.90E-03 |
| R^2^_C_ | 0.841 | 0.941 | 0.973 | 0.984 | 0.972 | 0.950 | 0.957 |
| R^2^_CV_ | 0.254 | 0.828 | 0.855 | 0.752 | 0.932 | 0.843 | 0.894 |
|  | Fe^3+^ | | | | | | |
| RMSEC | 9.09E-04 | 4.56E-04 | 3.40E-04 | 4.73E-04 | 3.87E-04 | 3.94E-04 | 3.93E-04 |
| RMSECV | 1.48E-03 | 6.39E-04 | 6.93E-04 | 7.17E-04 | 7.26E-04 | 6.53E-04 | 6.71E-04 |
| R^2^_C_ | 0.879 | 0.970 | 0.983 | 0.967 | 0.978 | 0.977 | 0.978 |
| R^2^_CV_ | 0.715 | 0.942 | 0.932 | 0.928 | 0.925 | 0.939 | 0.935 |
|  | Hg^2+^ | | | | | | |
| RMSEC | 9.37E-06 | 9.55E-06 | 9.13E-06 | 9.73E-06 | 9.32E-06 | 9.97E-06 | 9.43E-06 |
| RMSECV | 2.54E-05 | 2.01E-05 | 2.97E-05 | 2.82E-05 | 2.14E-05 | 2.25E-05 | 1.88E-05 |
| R^2^_C_ | 0.992 | 0.991 | 0.992 | 0.991 | 0.992 | 0.990 | 0.991 |
| R^2^_CV_ | 0.946 | 0.962 | 0.917 | 0.928 | 0.958 | 0.952 | 0.966 |
|  | Ni^2+^ | | | | | | |
| RMSEC | 9.84E-02 | 1.17E-02 | 1.21E-02 | 1.33E-02 | 1.67E-02 | 1.77E-02 | 1.21E-02 |
| RMSECV | 2.71E-01 | 4.82E-02 | 9.05E-02 | 2.50E-02 | 7.51E-02 | 7.45E-02 | 5.56E-02 |
| R^2^_C_ | 0.799 | 0.997 | 0.997 | 0.996 | 0.994 | 0.993 | 0.997 |
| R^2^_CV_ | 0.187 | 0.959 | 0.843 | 0.990 | 0.888 | 0.886 | 0.938 |
|  | Pb^2+^ | | | | | | |
| RMSEC | 2.11E-05 | 1.35E-05 | 1.70E-05 | 1.40E-05 | 1.37E-05 | 1.23E-05 | 1.37E-05 |
| RMSECV | 4.47E-05 | 1.87E-05 | 3.40E-05 | 2.70E-05 | 3.24E-05 | 2.34E-05 | 2.16E-05 |
| R^2^_C_ | 0.966 | 0.986 | 0.978 | 0.985 | 0.986 | 0.989 | 0.986 |
| R^2^_CV_ | 0.883 | 0.974 | 0.914 | 0.955 | 0.938 | 0.959 | 0.966 |
|  | Zn^2+^ | | | | | | |
| RMSEC | 4.40E-02 | 2.07E-02 | 2.58E-02 | 1.99E-02 | 2.28E-02 | 2.58E-02 | 2.27E-02 |
| RMSECV | 8.40E-02 | 4.45E-02 | 5.66E-02 | 4.92E-02 | 4.78E-02 | 5.53E-02 | 4.98E-02 |
| R^2^_C_ | 0.940 | 0.987 | 0.979 | 0.988 | 0.984 | 0.979 | 0.984 |
| R^2^_CV_ | 0.783 | 0.943 | 0.902 | 0.929 | 0.931 | 0.906 | 0.925 |

Note: the RMSEC and RMSECV values are expressed in mmol L^-1^.

**Table S3.** Confusion matrix for PLS-DA metal ions discrimination model, using spectral region R1, 9 LVs for validation data, and prior mean centering of PL data.

| Real metals ions | Predicted metal ions | | | | | | | | | | |
| --- | --- | --- | --- | --- | --- | --- | --- | --- | --- | --- | --- |
|  | Ag^+^ | Ca^2+^ | Cd^2+^ | Cu^2+^ | Fe^2+^ | Fe^3+^ | Hg^2+^ | Ni^2+^ | Pb^2+^ | Zn^2+^ | Al^3+^ |
| Ag^+^ | $\frac{3}{6}$ | 0 | 0 | $\frac{1}{6}$ | 0 | 0 | 0 | $\frac{1}{6}$ | 0 | $\frac{1}{6}$ | 0 |
| Ca^2+^ | 0 | $\frac{3}{5}$ | 0 | $\frac{2}{5}$ | 0 | 0 | 0 | 0 | 0 | 0 | 0 |
| Cd^2+^ | 0 | $\frac{1}{3}$ | 0 | 0 | $\frac{1}{3}$ | 0 | 0 | $\frac{1}{3}$ | 0 | 0 | 0 |
| Cu^2+^ | 0 | 0 | 0 | $\frac{1}{5}$ | 0 | $\frac{1}{5}$ | 0 | $\frac{2}{5}$ | 0 | 0 | $\frac{1}{5}$ |
| Fe^2+^ | 0 | 0 | 0 | 0 | $\frac{4}{6}$ | 0 | 0 | $\frac{2}{6}$ | 0 | 0 | 0 |
| Fe^3+^ | 0 | 0 | 0 | 0 | 0 | $\frac{6}{8}$ | $\frac{1}{8}$ | 0 | 0 | $\frac{1}{8}$ | 0 |
| Hg^2+^ | 0 | 0 | 0 | 0 | 0 | 0 | $\frac{3}{5}$ | $\frac{2}{5}$ | 0 | 0 | 0 |
| Ni^2+^ | 0 | 0 | 0 | 0 | $\frac{1}{7}$ | 0 | 0 | $\frac{6}{7}$ | 0 | 0 | 0 |
| Pb^2+^ | $\frac{1}{7}$ | 0 | 0 | 0 | $\frac{1}{7}$ | $\frac{1}{7}$ | $\frac{1}{7}$ | 0 | $\frac{2}{7}$ | $\frac{1}{7}$ | 0 |
| Zn^2+^ | $\frac{1}{6}$ | 0 | $\frac{1}{6}$ | $\frac{1}{6}$ | 0 | 0 | 0 | 0 | 0 | $\frac{3}{6}$ | 0 |
| Al^3+^ | 0 | 0 | 0 | 0 | 0 | 0 | 0 | 0 | 0 | 0 | $\frac{5}{5}$ |

**Table S4.** Confusion matrix for PLS-DA metal ions discrimination model, using spectral region R2, with 9 LVs for validation data, and prior mean centering of PL data.

| Real metals ions | Predicted metal ions | | | | | | | | | | |
| --- | --- | --- | --- | --- | --- | --- | --- | --- | --- | --- | --- |
|  | Ag^+^ | Ca^2+^ | Cd^2+^ | Cu^2+^ | Fe^2+^ | Fe^3+^ | Hg^2+^ | Ni^2+^ | Pb^2+^ | Zn^2+^ | Al^3+^ |
| Ag^+^ | $\frac{5}{6}$ | 0 | 0 | 0 | 0 | $\frac{1}{6}$ | 0 | 0 | 0 | 0 | 0 |
| Ca^2+^ | 0 | $\frac{4}{5}$ | 0 | 0 | 0 | 0 | 0 | $\frac{1}{5}$ | 0 | 0 | 0 |
| Cd^2+^ | 0 | $\frac{1}{3}$ | 0 | 0 | 0 | 0 | 0 | $\frac{1}{3}$ | 0 | $\frac{1}{3}$ | 0 |
| Cu^2+^ | 0 | 0 | 0 | $\frac{2}{5}$ | 0 | $\frac{1}{5}$ | $\frac{1}{5}$ | 0 | $\frac{1}{5}$ | 0 | 0 |
| Fe^2+^ | 0 | 0 | 0 | 0 | $\frac{3}{6}$ | $\frac{1}{6}$ | 0 | 0 | 0 | $\frac{2}{6}$ | 0 |
| Fe^3+^ | 0 | $\frac{1}{8}$ | 0 | 0 | 0 | $\frac{4}{8}$ | $\frac{1}{8}$ | 0 | 0 | $\frac{2}{8}$ | 0 |
| Hg^2+^ | 0 | 0 | 0 | 0 | $\frac{1}{5}$ | 0 | $\frac{1}{5}$ | $\frac{2}{5}$ | $\frac{1}{5}$ | 0 | 0 |
| Ni^2+^ | 0 | 0 | $\frac{1}{7}$ | 0 | $\frac{2}{7}$ | 0 | $\frac{1}{7}$ | $\frac{3}{7}$ | 0 | 0 | 0 |
| Pb^2+^ | 0 | 0 | 0 | 0 | 0 | 0 | 0 | 0 | $\frac{7}{7}$ | 0 | 0 |
| Zn^2+^ | 0 | $\frac{2}{6}$ | $\frac{1}{6}$ | 0 | 0 | $\frac{1}{6}$ | 0 | 0 | 0 | $\frac{2}{6}$ | 0 |
| Al^3+^ | 0 | 0 | 0 | 0 | 0 | 0 | $\frac{2}{5}$ | 0 | 0 | 0 | $\frac{3}{5}$ |

**Table S5.** Confusion matrix for PLS-DA metal ions discrimination model using spectral region R3, with 9 LVs for validation data, and prior mean centering of PL data.

| Real metals ions | Predicted metal ions | | | | | | | | | | |
| --- | --- | --- | --- | --- | --- | --- | --- | --- | --- | --- | --- |
|  | Ag^+^ | Ca^2+^ | Cd^2+^ | Cu^2+^ | Fe^2+^ | Fe^3+^ | Hg^2+^ | Ni^2+^ | Pb^2+^ | Zn^2+^ | Al^3+^ |
| Ag^+^ | $\frac{5}{6}$ | 0 | 0 | 0 | 0 | 0 | 0 | 0 | $\frac{1}{6}$ | 0 | 0 |
| Ca^2+^ | 0 | $\frac{5}{5}$ | 0 | 0 | 0 | 0 | 0 | 0 | 0 | 0 | 0 |
| Cd^2+^ | 0 | 0 | $\frac{2}{3}$ | 0 | 0 | 0 | 0 | 0 | $\frac{1}{3}$ | 0 | 0 |
| Cu^2+^ | 0 | 0 | 0 | $\frac{3}{5}$ | 0 | $\frac{2}{5}$ | 0 | 0 | 0 | 0 | 0 |
| Fe^2+^ | 0 | 0 | 0 | 0 | $\frac{4}{6}$ | 0 | $\frac{2}{6}$ | 0 | 0 | 0 | 0 |
| Fe^3+^ | 0 | $\frac{1}{8}$ | 0 | $\frac{1}{8}$ | 0 | $\frac{6}{8}$ | 0 | 0 | 0 | 0 | 0 |
| Hg^2+^ | 0 | 0 | 0 | 0 | 0 | $\frac{3}{5}$ | $\frac{2}{5}$ | 0 | 0 | 0 | 0 |
| Ni^2+^ | 0 | 0 | 0 | 0 | 0 | $\frac{1}{7}$ | $\frac{2}{7}$ | $\frac{3}{7}$ | $\frac{1}{7}$ | 0 | 0 |
| Pb^2+^ | 0 | 0 | 0 | 0 | 0 | 0 | 0 | $\frac{1}{7}$ | $\frac{6}{7}$ | 0 | 0 |
| Zn^2+^ | 0 | 0 | 0 | 0 | 0 | 0 | 0 | $\frac{1}{6}$ | 0 | $\frac{5}{6}$ | 0 |
| Al^3+^ | 0 | 0 | 0 | 0 | 0 | 0 | 0 | 0 | 0 | 0 | $\frac{5}{5}$ |

**Table S6.** Confusion matrix for PLS-DA metal ions discrimination model using spectral regions R1 + R2, with 9 LVs for validation data, and prior mean centering of PL data.

| Real metals ions | Predicted metal ions | | | | | | | | | | |
| --- | --- | --- | --- | --- | --- | --- | --- | --- | --- | --- | --- |
|  | Ag^+^ | Ca^2+^ | Cd^2+^ | Cu^2+^ | Fe^2+^ | Fe^3+^ | Hg^2+^ | Ni^2+^ | Pb^2+^ | Zn^2+^ | Al^3+^ |
| Ag^+^ | $\frac{6}{6}$ | 0 | 0 | 0 | 0 | 0 | 0 | 0 | 0 | 0 | 0 |
| Ca^2+^ | 0 | $\frac{4}{5}$ | 0 | 0 | 0 | 0 | 0 | $\frac{1}{5}$ | 0 | 0 | 0 |
| Cd^2+^ | 0 | $\frac{2}{3}$ | $\frac{1}{3}$ | 0 | 0 | 0 | 0 | 0 | 0 | 0 | 0 |
| Cu^2+^ | 0 | 0 | 0 | $\frac{2}{5}$ | 0 | $\frac{1}{5}$ | 0 | $\frac{1}{5}$ | 0 | 0 | $\frac{1}{5}$ |
| Fe^2+^ | 0 | 0 | 0 | 0 | $\frac{6}{6}$ | 0 | 0 | 0 | 0 | 0 | 0 |
| Fe^3+^ | 0 | 0 | 0 | 0 | 0 | $\frac{7}{8}$ | 0 | 0 | 0 | $\frac{1}{8}$ | 0 |
| Hg^2+^ | 0 | 0 | 0 | $\frac{1}{5}$ | 0 | 0 | $\frac{3}{5}$ | 0 | $\frac{1}{5}$ | 0 | 0 |
| Ni^2+^ | 0 | 0 | 0 | 0 | $\frac{1}{7}$ | 0 | 0 | $\frac{6}{7}$ | 0 | 0 | 0 |
| Pb^2+^ | 0 | 0 | 0 | 0 | 0 | 0 | 0 | 0 | $\frac{7}{7}$ | 0 | 0 |
| Zn^2+^ | 0 | 0 | 0 | 0 | 0 | 0 | 0 | 0 | 0 | $\frac{6}{6}$ | 0 |
| Al^3+^ | 0 | 0 | 0 | 0 | 0 | 0 | 0 | 0 | 0 | 0 | $\frac{5}{5}$ |

**Table S7.** Confusion matrix for PLS-DA metal ions discrimination model using spectral regions R1 + R3, with 9 LVs for validation data, and prior mean centering of PL data.

| Real metals ions | Predicted metal ions | | | | | | | | | | |
| --- | --- | --- | --- | --- | --- | --- | --- | --- | --- | --- | --- |
|  | Ag^+^ | Ca^2+^ | Cd^2+^ | Cu^2+^ | Fe^2+^ | Fe^3+^ | Hg^2+^ | Ni^2+^ | Pb^2+^ | Zn^2+^ | Al^3+^ |
| Ag^+^ | $\frac{6}{6}$ | 0 | 0 | 0 | 0 | 0 | 0 | 0 | 0 | 0 | 0 |
| Ca^2+^ | 0 | $\frac{4}{5}$ | 0 | 0 | 0 | 0 | 0 | $\frac{1}{5}$ | 0 | 0 | 0 |
| Cd^2+^ | 0 | 0 | $\frac{2}{3}$ | $\frac{1}{3}$ | 0 | 0 | 0 | 0 | 0 | 0 | 0 |
| Cu^2+^ | $\frac{1}{5}$ | 0 | 0 | $\frac{2}{5}$ | 0 | $\frac{1}{5}$ | 0 | 0 | 0 | 0 | $\frac{1}{5}$ |
| Fe^2+^ | 0 | 0 | 0 | 0 | $\frac{6}{6}$ | 0 | 0 | 0 | 0 | 0 | 0 |
| Fe^3+^ | 0 | 0 | 0 | 0 | 0 | $\frac{7}{8}$ | $\frac{1}{8}$ | 0 | 0 | 0 | 0 |
| Hg^2+^ | 0 | 0 | 0 | 0 | 0 | 0 | $\frac{4}{5}$ | $\frac{1}{5}$ | 0 | 0 | 0 |
| Ni^2+^ | 0 | 0 | 0 | 0 | $\frac{2}{7}$ | 0 | 0 | $\frac{5}{7}$ | 0 | 0 | 0 |
| Pb^2+^ | 0 | 0 | 0 | 0 | 0 | 0 | 0 | 0 | $\frac{7}{7}$ | 0 | 0 |
| Zn^2+^ | 0 | $\frac{1}{6}$ | 0 | 0 | 0 | 0 | 0 | 0 | 0 | $\frac{5}{6}$ | 0 |
| Al^3+^ | 0 | 0 | 0 | 0 | 0 | 0 | 0 | 0 | 0 | 0 | $\frac{5}{5}$ |

**Table S8.** Confusion matrix for PLS-DA metal ions discrimination model using spectral regions R2 + R3, with 9 LVs for validation data, and prior mean centering of PL data.

| Real metals ions | Predicted metal ions | | | | | | | | | | |
| --- | --- | --- | --- | --- | --- | --- | --- | --- | --- | --- | --- |
|  | Ag^+^ | Ca^2+^ | Cd^2+^ | Cu^2+^ | Fe^2+^ | Fe^3+^ | Hg^2+^ | Ni^2+^ | Pb^2+^ | Zn^2+^ | Al^3+^ |
| Ag^+^ | $\frac{6}{6}$ | 0 | 0 | 0 | 0 | 0 | 0 | 0 | 0 | 0 | 0 |
| Ca^2+^ | 0 | $\frac{4}{5}$ | 0 | 0 | 0 | 0 | 0 | $\frac{1}{5}$ | 0 | 0 | 0 |
| Cd^2+^ | 0 | 0 | $\frac{3}{3}$ | 0 | 0 | 0 | 0 | 0 | 0 | 0 | 0 |
| Cu^2+^ | 0 | 0 | 0 | $\frac{3}{5}$ | 0 | $\frac{1}{5}$ | 0 | 0 | $\frac{1}{5}$ | 0 | 0 |
| Fe^2+^ | 0 | 0 | 0 | 0 | $\frac{6}{6}$ | 0 | 0 | 0 | 0 | 0 | 0 |
| Fe^3+^ | 0 | 0 | 0 | 0 | 0 | $\frac{7}{8}$ | $\frac{1}{8}$ | 0 | 0 | 0 | 0 |
| Hg^2+^ | 0 | 0 | 0 | 0 | 0 | 0 | $\frac{5}{5}$ | 0 | 0 | 0 | 0 |
| Ni^2+^ | 0 | 0 | 0 | 0 | 0 | 0 | 0 | $\frac{7}{7}$ | 0 | 0 | 0 |
| Pb^2+^ | 0 | 0 | 0 | 0 | 0 | 0 | 0 | 0 | $\frac{7}{7}$ | 0 | 0 |
| Zn^2+^ | 0 | 0 | 0 | 0 | 0 | $\frac{1}{6}$ | 0 | 0 | 0 | $\frac{5}{6}$ | 0 |
| Al^3+^ | 0 | 0 | 0 | 0 | 0 | 0 | 0 | $\frac{1}{5}$ | 0 | 0 | $\frac{4}{5}$ |

**Table S9.** Calibration and validation results for the PLS (first-order data) and U-PLS (second-order data) considering Hg^2+^ with Pb^2+^ mixtures. Note that the PL data were previously mean centered.

|  | First experiment | | Second experiment | | Third experiment | |
| --- | --- | --- | --- | --- | --- | --- |
|  | First-order data - PLS | | | | | |
|  | Hg^2+^ | Pb^2+^ | Hg^2+^ | Pb^2+^ | Hg^2+^ | Pb^2+^ |
| LV | 4 | 1 | 4 | 2 | 4 | 2 |
| RMSECV* | 1.35E-05 | 3.72E-05 | 1.44E-05 | 3.12E-05 | 1.71E-05 | 3.42E-05 |
| R^2^_CV_ | 0.879 | 0.165 | 0.867 | 0.828 | 0.873 | 0.783 |
| RMSEP* | 9.76E-06 | 2.203-05 | 1.48E-05 | 3.08E-05 | 8.99E-06 | 1.87E-05 |
| R^2^_P_ | 0.868 | 0.780 | 0.794 | 0.834 | 0.956 | 0.955 |
|  | Second-order data - U-PLS | | | | | |
| LV | 4 | 3 | 2 | 2 | 2 | 4 |
| RMSECV* | 1.03E-05 | 2.92E-05 | 1.83E-05 | 3.33E-05 | 1.56E-05 | 1.89E-05 |
| R^2^_CV_ | 0.931 | 0.658 | 0.748 | 0.797 | 0.893 | 0.941 |
| RMSEP* | 6.28E-06 | 2.62E-05 | 1.41E-05 | 2.24E-05 | 8.17E-06 | 1.32E-05 |
| R^2^_P_ | 0.964 | 0.659 | 0.902 | 0.897 | 0.961 | 0.975 |

* values expressed in mmol L^-1^.

**Table S10.** Calibration and validation results for the PLS (first-order data) and U-PLS (second-order data) considering Al^3+^ with Hg^2+^ mixtures. Note that the PL data were previously mean centered.

|  | First experiment | | Second experiment | | Third experiment | |
| --- | --- | --- | --- | --- | --- | --- |
|  | First-order data - PLS | | | | | |
|  | Al^3+^ | Hg^2+^ | Al^3+^ | Hg^2+^ | Al^3+^ | Hg^2+^ |
| LV | 4 | 1 | 1 | 1 | 1 | 1 |
| RMSECV* | 2.54E-05 | 2.18E-05 | 4.02E-05 | 3.29E-05 | 1.62E-05 | 1.65E-05 |
| R^2^_CV_ | 0.858 | 0.636 | 0.746 | 0.512 | 0.930 | 0.907 |
| RMSEP* | 3.35E-05 | 1.62E-05 | 1.93E-05 | 2.78E-05 | 2.54E-05 | 3.23E-05 |
| R^2^_P_ | 0.795 | 0.809 | 0.930 | 0.669 | 0.879 | 0.798 |
|  | Second-order data - U-PLS | | | | | |
| LV | 3 | 1 | 1 | 1 | 1 | 3 |
| RMSECV* | 3.90E-05 | 1.82E-05 | 5.97E-10 | 3.21E-05 | 1.69E-05 | 1.13E-05 |
| R^2^_CV_ | 0.776 | 0.801 | 0.868 | 0.569 | 0.922 | 0.959 |
| RMSEP* | 2.85E-05 | 1.39E-05 | 3.13E-05 | 2.74E-05 | 2.18E-05 | 2.11E-05 |
| R^2^_P_ | 0.795 | 0.840 | 0.938 | 0.692 | 0.920 | 0.892 |

* values expressed in mmol L^-1^.


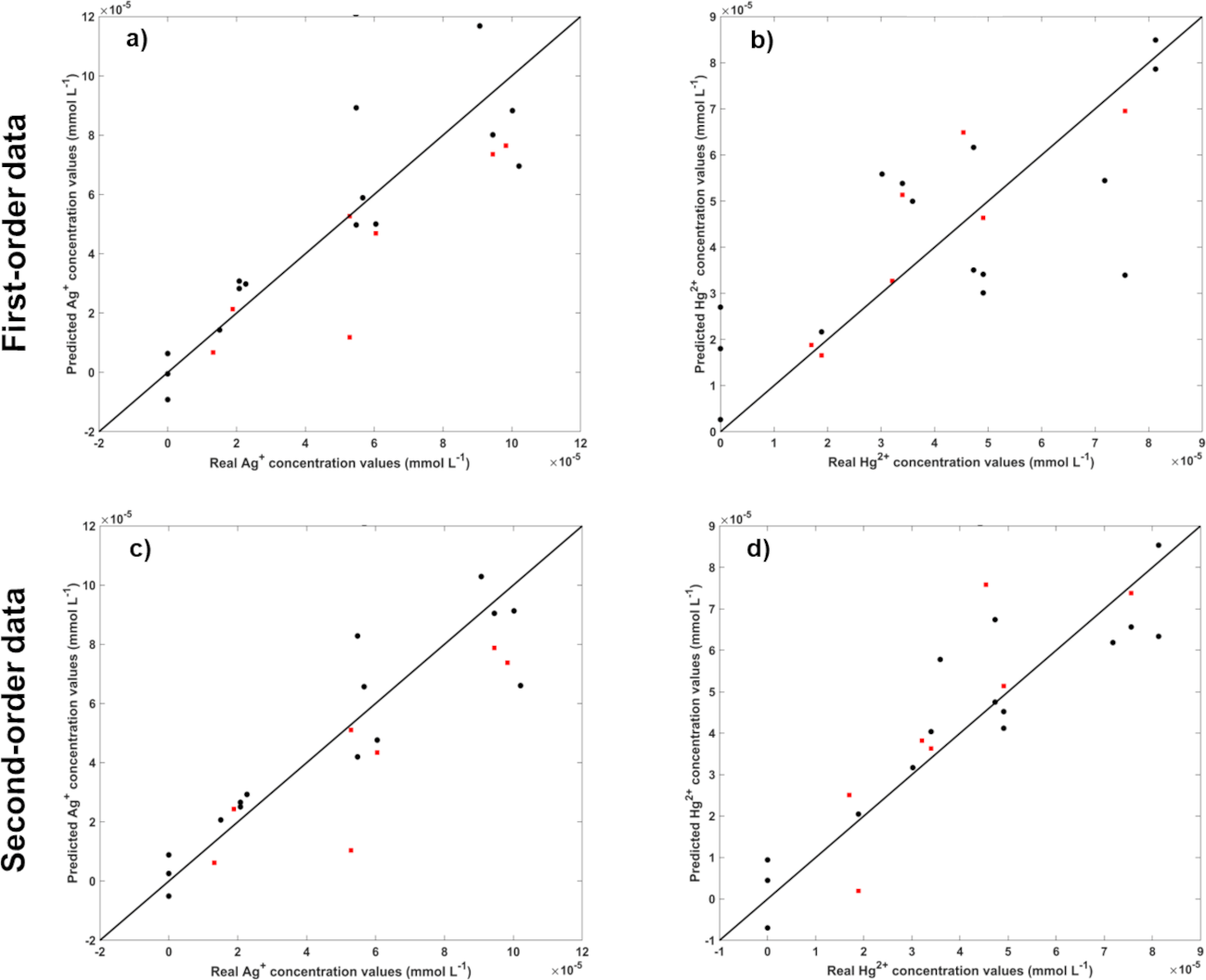


**Fig. S4.** Real values versus the predicted cross-validation (●) and validation (■) values obtained for PLS (a and b) and U-PLS (c and d) models considering the entire spectral range and the mixture of Ag^+^ (a and c) and Hg^2+^ (b and d). Note that PL data was mean-centered previously.

**Table S11.** Calibration and validation results for the PLS (first-order data) and U-PLS (second-order data) models considering Ag^+^ with Hg^2+^ mixture. Note that the PL data were previously mean centered.

|  | First-order data - PLS | | Second-order data - U-PLS | |
| --- | --- | --- | --- | --- |
|  | Ag^+^ | Hg^2+^ | Ag^+^ | Hg^2+^ |
| LV | 2 | 2 | 2 | 2 |
| RMSECV* | 1.58E-05 | 1.88E-05 | 1.41E-05 | 1.06E-05 |
| R^2^_CV_ | 0.905 | 0.733 | 0.923 | 0.922 |
| RMSEP* | 2.01E-05 | 1.02E-05 | 2.08E-05 | 1.38E-05 |
| R^2^_P_ | 0.890 | 0.879 | 0.877 | 0.848 |

* values expressed in mmol L^-1^.
